# Alternative strategies based on transgenic *Drosophila melanogaster* for the functional characterization of insect Ionotropic Receptors

**DOI:** 10.1186/s40659-025-00619-0

**Published:** 2025-06-09

**Authors:** Cristina M. Crava, William B. Walker, Alberto Maria Cattaneo

**Affiliations:** 1 University Institute of Biotechnology and Biomedicine, University of Valencia, 46100, Burjassot, Spain; 2 USDA-ARS Temperate Tree Fruit and Vegetable Research Unit, 5230 Konnowac Pass Road, Wapato, WA 98951 USA; 3 Chemical Ecology, Department of Plant Protection Biology, Swedish University of Agricultural Sciences, Växtskyddsvägen 3, 234 56, Lomma (Campus Alnarp), Sweden; 4 Benton Lab, Center for Integrative Genomics, University of Lausanne, Lausanne, Quartier Sorge, CH-1015 Switzerland

**Keywords:** Insect ionotropic receptors, Transgenic *Drosophila melanogaster*, Heterologous expression, Single sensillum recording, Functional characterization, Coeloconic sensilla ac4, Deorphanization

## Abstract

**Background:**

Insect Ionotropic Receptors (IRs) are a relatively uncharted territory. Some studies have documented IR activation by recording neuronal activity in situ, others by their heterologous expression in *Xenopus* oocytes or mis-expressing IRs from *Drosophila melanogaster* or from the related *D. sechellia* into the *D. melanogaster* “ionotropic receptor decoder” neuron, which lacks the endogenous tuning receptor subunit but expresses IR-coreceptors.

**Results:**

In this study, we first made use of *Drosophila* olfactory sensory neurons (OSNs) different from the “ionotropic receptor decoder”, demonstrating that by replacing or introducing IRs alongside the native *D. melanogaster* ones, functional heteromeric complexes can be formed. IR41a1 from the lepidopteran *Cydia pomonella* exhibits binding to polyamines and the IR75d from the dipteran *Drosophila suzukii* binds hexanoic acid. Secondly, expressing *D. suzukii*’s putative acid sensor IR64a into the “ionotropic receptor decoder” of *D. melanogaster* inhibits the response to the main activators of neighboring neurons from the same sensillum, despite that IR64a does not respond to acids. In situ hybridization on the antennae of *D. suzukii* unveils wide expression of IR64a in neurons proximal to the sacculus. Structural modeling analysis does not explain its absence of binding to acids; conversely, this approach identifies key amino acids features explaining the binding of hexanoic acid by IR75d. Finally, we have also explored alternative methods to heterologously express IRs based on Human Embryonic Kidney cells (HEK293). Despite observing correct expression of IRs in transfected cells through immunohistochemistry experiments, this approach did not achieve successful deorphanization of these receptors.

**Conclusion:**

Our findings highlight the potential use of *Drosophila* OSNs as a valuable tool for functional characterization of IRs from different insect species: for the first time, we have provided evidence of IR-functionalities within alternative OSNs from the *Drosophila*’s "ionotropic receptor decoder” neuron to functionally characterize and deorphanize IRs from lineages that are evolutionarily distant from the *D. melanogaster* subgroup, contributing to the understanding of chemosensory modalities in *D. suzukii* and *C. pomonella*, two globally significant agricultural pests. Additionally, the unsuccessful deorphanization in HEK cells highlights the complex requirements for IR functionality, supporting the use of *Drosophila* OSNs as a more suitable expression system.

**Supplementary Information:**

The online version contains supplementary material available at 10.1186/s40659-025-00619-0.

## Background

Ionotropic Receptors (IRs) are transmembrane chemoreceptors found in the peripheral sensory neurons of animals belonging to the superphylum Protostomia, which includes arthropods, nematodes, mollusks, annelids and other invertebrates [1–3]. These receptors share an evolutionary relationship to the Ionotropic Glutamate Receptors (iGluRs), which are an ancient class of ligand-gated ion channels involved in neuronal communication across the animal kingdom [4]. iGluRs are also present in a small number of prokaryotes [5, 6] and plants [7]. IRs were first discovered in *Drosophila melanogaster* [8], and subsequent research on these chemoreceptors has primarily focused on this species and a few mosquito species. Olfactory IRs function as ligand-gated ion channels, which allow the influx of cations into the cytoplasm and physiological activation of the olfactory sensory neuron (OSN) [9]. Each IR-gated ion channel consists of individual odor-specific tuning subunits and one or two broadly expressed co-receptors (IR25a, IR8a, and IR76b) [9–11]. Collectively, in *D. melanogaster*, the functional characterization of the tuning IR subunits has led to the identification of activators for a total of eighteen different subunits. These agonists include acids and amines, as well as other chemical activators such as sucrose, amino acids, calcium, and carbonation. In addition, some IRs respond to physical stimuli such as moisture, dryness, and cool temperatures [12].

Such deorphanization efforts have benefited from the genetic toolbox available for this species, which enables a variety of approaches. These include recording ligand-induced neuronal activity in situ in knocked out lines [13–16], functional imaging experiments [15, 17–19] or mis-expression of heterologous IRs in different OSNs [8–10, 19] or in the “ionotropic receptor decoder” neuron, which is an OSN lacking the endogenous tuning receptor subunit but expressing the IR8a coreceptor [10, 14, 20–22]. In contrast, such a wide array of genetic tools is not readily available for non-model species, slowing down the comprehension of the role of IRs outside *Drosophila*. So far, the methods used include knock down, which is not straightforward in many non-model species, in situ recording of neuronal activity [23] or the heterologous expression in *Xenopus laevis* oocytes [23–27]. This latter approach is a commonly used heterologous expression system for the electrophysiological characterization of iGluRs, and it has been the only method used so far for functional characterization of IRs from lineages outside Diptera species, specifically the lepidopteran *Agrotis segetum* [27] and the wasp *Microplitis mediator* [26]. The use of *Xenopus* relies on a single cell system extremely different from the native OSN environment, which imposes significant limitations on our understanding of the activation and pharmacology of the expressed receptor [28].

An interesting in vivo alternative is the above-mentioned “ionotropic receptor decoder”, which closely resembles the *Drosophila* “empty neuron” system. The latter has been extensively used for functional characterization of another class of insect olfactory receptors, the odorant receptors (ORs), in insect species both within and beyond dipterans [29]. However, the “ionotropic receptor decoder” has thus far been successfully used to deorphanize IR tuning subunits only from *D. melanogaster* and a closely related species from the melanogaster subgroup, *Drosophila sechellia* [10, 14, 20–22], and it has not been tested for IR subunits from other evolutionary lineages. This raises the possibility that the system may not be effective for more distantly related species, such as Lepidoptera, due to differences between co-receptors.

**Fig. 1:**
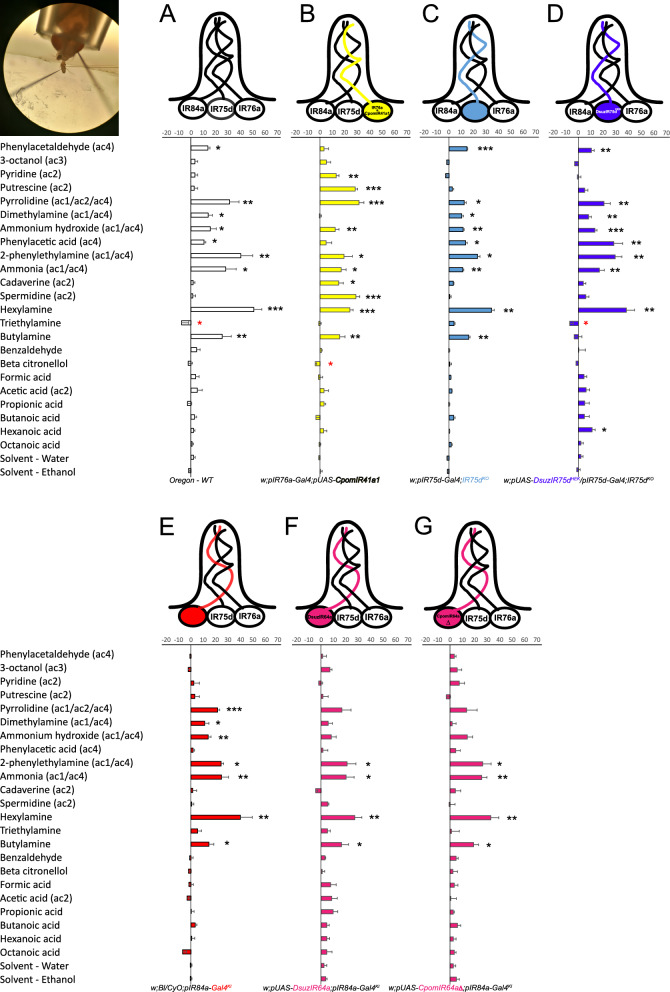
Odor responses of *D. melanogaster* ac4 sensilla expressing heterologous *C. pomonella* and *D. suzukii* IRs. Bars represent the mean evoked responses (± SEM) of ac4 sensilla (representing the summed-activities of the IR76a-expressing, IR75d-expressing, and IR84a-expressing neurons) to a panel of 23 odors. Asterisks indicate compounds enhancing significant increase (black) or decrease (red) in spike frequencies (Δspikes/0.5 s) when compared with their respective solvent either by a paired T-test or by a Wilcoxon Signed Rank Test (* *p* < 0.05; ** 0.001 < *p* < 0.01; *** *p* < 0.001, N = 5–9 depending on the experiment). Acronyms in parenthesis (ac1-4) indicate whether the corresponding compound is expected to be active on a specific type of coeloconic sensilla, based on Silbering et al. [17] and DoOR database [35, 36] (Table 1). (**A**) Odor response profiles of ac4 sensilla from wild-type *D. melanogaster* Oregon-C (white). (**B**) Odor response profiles from the transgenic line expressing CpomIR41a1 of *C. pomonella* in DmelIR76a-expressing neurons [*w;IR76a-Gal4;UAS-CpomIR41a1*]. (**C**) Odor response profiles of ac4 sensilla from a *D. melanogaster* transgenic line not expressing the endogenous DmelIR75d subunits [*w;IR75d-Gal4;IR75d*^*KO*^]. (**D**) Odor response profiles of transgenic line not expressing the endogenous DmelIR75d subunits and expressing DsuzIR75d^HEK^ [*w;IR75d-Gal4/UAS-DsuzIR75d*^*HEK*^*;IR75d*^*KO*^]. (**E**) Odor response profiles of ac4 sensilla from *D. melanogaster IR84a*^*KO*^ flies not expressing endogenous DmelIR84a subunit [*w;Bl/CyO;IR84a-Gal4*^*KI*^]. (**F**) Odor response profiles of ac4 sensilla from a transgenic line where DsuzIR64a replaced DmelIR84a [*w;UAS-DsuzIR64a;IR84a-Gal4*]. (**G**)—Odor response profiles of ac4 sensilla from a transgenic line where the truncated CpomIR64a∆ replaced DmelIR84a[*w;UAS-CpomIR64a∆;IR84a-Gal4*^*KI*^]. Top left: schematic figure panel that illustrates how the recordings are done. Note: electrodes are visible as black shadows directed on the insect, where the recording electrode is on the left, laying on the antennae, while the reference electrode is on the right, penetrating the fly’s eye

In this study, our aim is to explore alternative methods for heterologous expression of IRs, with the goal of providing new tools for comprehensive functional characterization of insect IRs beyond *Drosophila*. Specifically, we focused on two agricultural pests: the lepidopteran codling moth, *Cydia pomonella,* and one drosophilid species outside the melanogaster species subgroup, the spotted winged drosophila, *Drosophila suzukii*. In both species, we selected IRs orthologous to *D. melanogaster* DmelIR41a, DmellIR75d, and DmelIR64a, whose agonists are already known [13, 17–19]. To achieve this goal, we explored the use of two systems: an in vivo expression system using several OSNs housed in the ac4 sensillum of *D. melanogaster* as target and an ex vivo heterologous system utilizing Human Embryonic Kidney (HEK293T) cells. The ac4 sensillum houses three OSNs, each expressing one of three tuning subunits: DmelIR84a, DmelIR75d, and DmelIR76a [17] (Fig. 1A). The responses of both DmelIR75d and DmelIR76a are dependent on co-receptor DmelIR25a, although the latter also requires the expression of the DmelIR76b co-receptor subunit for accurate detection of polyamines [10, 17]. On the contrary, DmelIR84a subunit forms a heterotetramer with DmelIR8a co-receptor [10, 14, 17]. We substituted the *D. melanogaster* tuning receptors with *D. suzukii* IRs or expressed the *C. pomonella* tuning receptor alongside the *D. melanogaster* native receptors, demonstrating that both approaches result in a functional heteromeric complex. In contrast, our attempts to express functional IRs in HEK293T cells were unfruitful. Our results provide the first successful examples of heterologous in vivo expression of IRs from distant species in *Drosophila*, either by the use of a novel “ionotropic receptor decoder” or by expressing a foreign IR alongside a native one. This breakthrough opens up new possibilities for the application of these powerful tools in the deorphanization of ionotropic receptors of non-model insect species.

## Methods

### Fly strains and rearing

Wild-type and transgenic *D. melanogaster* were maintained on a sugar-yeast-cornmeal diet (https://bdsc.indiana.edu/information/recipes/bloomfood.html) at room temperature (25 ± 2 °C), and a relative humidity of 50 ± 5% under 12:12 light:dark photoperiod.

The Oregon-R strain of the fruit fly *D. melanogaster* was used as a wild-type (WT) strain. Transgenic *D. melanogaster* lines used in this study were: IR84a^GAL4^ (RRID: BDSC_41750) [14], IR75d-GAL4 (RRID: BDSC_41729) [17], IR75d^KO^ (RRID: BDSC_24205) [30], and IR76a-GAL4 (RRID: BDSC_98405) [17].

### Amplification and cloning of *C. pomonella* and *D. suzukii* IRs

The open reading frame (ORF) of CpomIR41a1 was obtained from transcriptomic analysis of *C. pomonella* antennal-expressed mRNAs [31, 32], whereas *D. suzukii* CDSs from a manually curated genome annotation [33]. Total RNA from *C. pomonella* was extracted and purified with a combined approach of TRIzol-based extraction followed by RNeasy^®^ Mini spin column purification (Qiagen, Venlo, Netherlands), and later retro-transcribed to cDNA using RT-for-PCR kit (Invitrogen, Life technologies, Grand Island, NY, USA). To clone *C. pomonella* IRs, we used specific primers (Supplementary Table S1) for amplifying the complete ORF encoding CpomIR41a1 or the truncated ORF of CpomIR64a (UniProt A0A0V0J232, from now on, CpomIR64a∆) adding upstream *attB1* and *attB2* sites suitable for BP-clonase-recombination (Gateway Technology, Invitrogen). The same method was also used to integrate *attB* regions upstream and downstream the complete DsuzIR75d ORF, which was amplified from the human HEK-codon-optimized sequence of DsuzIR75d cloned into pcDNA3.1 produced by GeneArt™ gene synthesis service (Thermo Fisher Scientific, Waltham, MA, USA) (from now on, DsuzIR75d^HEK^). All amplifications were run using a temperature program of 98 °C for 5 min followed by 45 cycles of 98 °C for 1 min, annealing step of 1 min (°C according to the primer pair used) and elongation at 72 °C for 70–90 s, with a final elongation step of 68 °C for 7 min. Purified PCR products were then cloned into the pDONR221 plasmid (Invitrogen). In the case of DsuzIR64a, the complete ORF cloned into pDONR221, was obtained by in vitro gene synthesis (GeneArt Thermo Fisher). The cassettes with inserts were then transferred to the destination vector (UASg-HA.*attB*, constructed by E. Furger and J. Bischof, kindly provided by the Basler group, Zürich), using the Gateway LR Clonase II kit (Invitrogen). Integrity and orientation of inserts was checked by Sanger sequencing before generating transgenic *D. melanogaster* lines.

### Heterologous expression in *D. melanogaster*

Best Gene (Chino Hills, CA, USA) generated transformed lines by PhiC31 standard integration. UAS-CpomIR41a1 integrated on the 3rd chromosome using attP2 strain (RRID: BDSC_8622), while UAS-DsuzIR75d^HEK^, UAS-DsuzIR64a, and UAS-CpomIR64a∆ integrated on the 2nd chromosome using the attp40 strain (RRID: BDSC_36304). Crossings were then performed as shown in Supplementary Figure S1 In brief, we used balancer lines in accordance with procedures already published from our lab [29]. To generate CpomIR41a1 expressing line, we used IR76a-GAL4 parental lines upon balancing. To generate DsuzIR75d^HEK^ expressing line, we crossed the IR75d-GAL4 line (*w;IR75d-Gal4;TM2/TM6B*) and the IR75d^KO^ line (*w[1118];Mi{ET}IR75d[MB04616]*) to obtain a line not expressing endogenous IR75d (*w;IR75d-Gal4;IR75d*^*KO*^*),* which was further crossed with the *w;UAS-DsuzIR75d*^*HEK*^*;IR75d*^*KO*^ line produced in this study. To generate DsuzIR64a and CpomIR64a∆ expressing lines, we used a IR84a-Gal4 knock-in parental line upon balancing (*w;Bl/CyO;IR84a-Gal4*^*KIKI*^). In this way, Gal4-knock-in replacing IR84a made an IR84a-knock-out out of it. The final strains used in SSR experiments had the following genotypes: CpomIR41a1-expressing line: *w;IR76a-Gal4;UAS-CpomIR41a1 *;DsuzIR75d-expressing line: *w;UAS-DsuzIR75d*^*HEK*^*/IR75d-Gal4;IR75d*^*KO *^; DsuzIR64a-expressing line: *w;UAS-DsuzIR64a;IR84a-Gal4*^*KI *^; CpomIR64a∆-expressing line: *w;UAS-CpomIR64a*∆*;IR84a-Gal4*.

### Single sensillum recordings

CpomIR41a1, DsuzIR75d, DsuzIR64a and CpomIR64a∆ expressed in the neurons of coeloconic sensilla ac4 were tested through single sensillum recordings (SSRs) as previously described [34]. In brief, 3-to-8-day old male flies were inserted in 100 μL pipette tips with only the top half of the head protruding. For each insect, the right antenna of the animal was gently pushed with a glass capillary against a piece of glass. This piece of glass and the pipette tip were fixed with dental wax on a microscope slide. Electrolytically sharpened tungsten electrodes (Harvard Apparatus Ltd, Edenbridge, United Kingdom) were used to penetrate the insect's body: the reference electrode was manually inserted in the right eye of the fly, while the recording electrode was maneuvered with a DC-3 K micromanipulator equipped with a PM-10 piezo translator (Märzhäuser Wetzler GmbH, Wetzler, Germany) and inserted into the sensillum to be recorded. Signals coming from sensory neurons were amplified 10 times with a probe (INR-02, Syntech, Hilversum, the Netherlands), digitally converted through an IDAC-4-USB (Syntech) interface and visualized and analyzed with the software Autospike v. 3.4 (Syntech). To carry the odorant stimulus as well as prevent antennal dryness and minimize the influence of background odors from the environment, a constant humidified flow of 2.5 L/min charcoal-filtered air was delivered through a glass tube and directed to the preparation.

The panel of the tested 23 ligands (Table 1) included control ligands, aimed to indicate choice of the correct sensillum among ac1-4 types [17, 18, 31–37]. Stimuli were diluted either in water or ethanol (Sigma Aldrich, St. Louis, MO-USA) depending on their solubility (Table 1) and then prepared as described by Silbering et al. [17], using 20 μL of 1% dilutions or 30 μL of 10 μg/μL for phenylacetic acid. Stimuli were then loaded on grade 1—20 mm circles filter paper (GE Healthcare Life Science, Little Chalfont, United Kingdom), previously inserted into glass Pasteur pipettes (VWR, Milan, Italy). To minimize possible effects from the solvent, pipettes were left at least 10 min after preparation under the fume hood for solvent evaporation. Subsequently, loaded pipettes were inserted into a side hole of the glass tube with the humidified airflow directed to the antennae and a puffing provided additional 2.5 mL air through the pipette for 0.5 s.

**Table 1:** Panel of ligands tested on transgenic *D. melanogaster*

Compound	Compound class	CAS	Molecular weight (g/mol)^a^	Vapor pressure (mmHg @ 20–25 °C)^a^	Density (g/mL)^a^	Moles in stimulus^b^	Solubility	Expected *ac* sensillum specificity^c^
Phenylacetaldehyde	Aromatic aldehyde	122–78-1	120.15	0.39	1.079	1.8 × 10^–6^	Water	ac4
1-octanol	Alcohol	111–87-5	130.23	0.0794	0.83	1.27 × 10^–6^	Ethanol	ac3
Pyridine	Tertiary amine	110–86-1	79.10	20.8	0.983	2.48 × 10^–6^	Ethanol	ac2
Putrescine	Diamine	110–60-1	88.15	2.33	0.877	2 × 10^–6^	Water	ac2
Pyrrolidine	Secondary amine	123–75-1	71.12	62.7	0.852	2.4 × 10^–6^	Water	ac1/ac2/ac4
Dimethylamine	Secondary amine	124–40-3	45.09	1520	0.670	2.97 × 10^–6^	Water	ac1/ac4
Ammonium hydroxide	Non-metal hydroxide	1336–21-6	35.05	2160	0.900	5.14 × 10^–6^	Water	ac1/ac4
Phenylacetic acid	Aromatic carboxylic acid	103–82-2	136.15	0.0038	1.081	2.38 × 10^–6^	Water	ac4
2-Phenylethylamine	Aromatic amine	64–04-0	121.18	0.23	0.964	1.59 × 10^–6^	Ethanol	ac1/ac4
Ammonia	Pnictogen hydride	7664–41-7	17.03	7500	0.682	8.01 × 10^–6^	Water	ac1/ac4
Cadaverine	Diamine	462–94-2	102.18	1.01	0.873	1.71 × 10^–6^	Water	ac2
Spermidine	Polyamine	124–20-9	145.25	0.001	0.925	1.27 × 10^–6^	Water	ac2
Hexylamine	Primary amine	111–26-2	101.19	7.95	0.77	1.52 × 10^–6^	Water	Unknown
Triethylamine	Tertiary amine	121–44-8	101.19	57.07	0.726	1.43 × 10^–6^	Water	Unknown
Butylamine	Primary amine	109–73-9	73.14	92.9	0.74	2.02 × 10^–6^	Water	Unknown
Benzaldehyde	Aromatic aldehyde	100–52-7	106.12	1.27	1.044	1.97 × 10^–6^	Ethanol	Unknown
β-citronellol	Monoterpenoid	106–22-9	157.27	0.02	0.857	1.09 × 10^–6^	Ethanol	Unknown
Formic acid	Carboxylic acid	64–18-6	46.03	36.477	1.22	5.3 × 10^–6^	Water	Unknown
Acetic acid	Carboxylic acid	64–19-7	60.05	15.7	1.049	3.49 × 10^–6^	Water	ac2
Propionic acid	Carboxylic acid	79–09-4	74.08	3.53	0.988	2.67 × 10^–6^	Water	ac2
Butanoic acid	Carboxylic acid	107–92-6	88.11	1.65	1.135	2.57 × 10^–6^	Water	Unknown
Hexanoic acid	Carboxylic acid	142–62-1	116.16	0.0435	0.929	1.6 × 10^–6^	Water	ac4
Octanoic acid	Carboxylic acid	124–07-2	144.21	0.0037	0.91	1.26 × 10^–6^	Water	No ac sensilla
Water	Oxygen hydride	7732–18-5	18.02	24.475	0.997	1.1 × 10^–6^	Water	Solvent
Ethanol	Primary alcohol	64–17-5	40.07	59.3	0.790	3.94 × 10^–6^	Ethanol	Solvent

^a^Data obtained from the database of odorant responses DoOR (http://neuro.uni-konstanz.de/DoOR/content/DoOR.php) and the good scents company (http://www.thegoodscentscompany.com/search2.html)

^**b**^Moles calculated for using 20 μL of 1% dilutions or 30 μL of 10 μg/μL for phenylacetic acid

^**c**^Based on Silbering et al. [17] and the database of odorant responses DoOR

The intensity of the response was quantified by counting all spikes recorded from an individual sensillum as conducted in Silbering et al. [17], because of the given difficulties in reliably distinguishing spikes from individual neurons [38]. Spike frequency was calculated by subtracting spikes counted for 0.5 s before the stimulus from the number of spikes counted for 0.5 s after the stimulus (∆spikes/0.5 s). Responses to compounds of the panel were obtained for 5 to 9 replicates, using a single insect as a replicate. Significant differences in spike counting were detected as previously done in Pettersson and Cattaneo [39]. In brief, spike frequencies for each tested compound were analyzed across replicates. Normality tests were performed using IBM SPSS Statistics software 29.0 (https://www.ibm.com/) to determine whether the data met parametric assumptions (Supplementary Table S2). Based on the results, both a parametric paired T-test and a non-parametric paired Wilcoxon Signed Rank Test [ɑ = 0.05] were conducted using the same software (Supplementary Data File 1). For each replicate, spike frequencies of each compound were compared with the spike frequencies of its respective solvent control (water/ethanol).

### Dose response SSR experiments

When testing dose-dependent responses of ac4 sensilla expressing CpomIR41a1 or DsuzIR75d, SSR method was adopted to perform dose–response experiments. Based on results from our SSR screening (Fig. 1), for CpomIR41a1-expressing ac4 sensilla we analyzed responses to 1,4-diaminobutane (putrescine) and N-(3-aminopropyl)butane-1,4-diamine (spermidine) along with hexylamine. For DsuzIR75d-expressing ac4 sensilla, responses to hexanoic acid were analyzed.

To test dose-responses, compounds were diluted in water at concentrations ranging from 0.1 to 3%. Then aliquots (1 to 30 μL depending on the experiment) were applied on 1–20 mm circles filter paper, in order to provide stimuli, in which neat ligands ranged from about 1 nL to about 1 μL (doses 0.1 to 90, Supplementary Data File 2). Spike frequencies were calculated as ∆spikes/0.5 s. Normalization for CpomIR41a1-expressing ac4 sensilla was based on the effect of the saturating doses of putrescine (DOSE 20), which was selected as the compound with the lower molecular weight (MW_putr.._ = 88.15 g/mol; MW_sperm._ = 145.25 g/mol) and the highest volatility (Vp_putr._ = 2.33 mmHg; Vp_sperm._ = 0.001 mmHg). Normalization for DsuzIR75d-expressing ac4 sensilla was based on the saturating DOSEs 10 or 20, depending on the experiment. Normalized data were analyzed by SigmaPlot 13.0 (Systat Software Inc., San Jose, CA, USA). Depending on the experiment, responses to selected compounds were obtained for 5–13 replicates, considering a replicate as a single insect (Supplementary Data File 2).

### Fluorescent in situ hybridization

Fluorescent *in situ* hybridization (FISH) was performed as reported in Cattaneo et al. [34]. Digoxigenin (DIG)-labelled probes were produced from linearized pDONR221-vectors containing DsuzIR64a and DsuzIR62a ORFs. This latter was used as negative control, and its complete ORF was amplified with primers (Supplementary Table S1) adding upstream *attB* sites and cloned in pDONR211 vector following the protocol described above. Linearized vectors were used as template for T7 RNA polymerase (Promega, Madison, WI, USA) synthesis integrating DIG-labeled ribonucleotides (BMB, Roche, Basel, Switzerland). As positive control, we used a Fluorescein-labeled probe targeting the olfactory receptor co-coreceptor (Orco), which was already described in a previous study [34]. FISH was carried out on whole mount antennae collected from male and female wild type adult insects of our rearing facility (FORMAS Swedish Research Council-project numbers 2011–390 and 2015–1221), adjusting protocols described by Saina & Benton [40] using a single probe for each experiment, Anti-Dig-Peroxidase (POD) or anti-Fluorescein-POD (Roche, Basel, Switzerland; http://www.roche.com/) depending on the probe used, and the Tyramide Signal Amplification (TSA) Plus-Cy5 System (Perkin Elmer, Inc., Waltham, MA, USA). Imaging was performed on a Zeiss confocal microscope LSM710 using a 40x immersion objective. DIG-labeled probes staining specific neurons were visualized setting Cy5-laser between 4 and 10% and calibrating gain in a range of 700–900. Staining was compared with male and female FISH-negative control probes for *DsuzIR62a*. Neuronal counting was performed using the cell-counter tool of Image J [41]. To identify differences between males and females, a parametric heteroscedastic Two-sample T-test was adopted [ɑ = 0.05], upon testing normality of the data and equality of their variances [ɑ = 0.05].

### Structural analysis

Since three-dimensional structures of the IRs from this study were not available, structural analysis were performed starting from structures from the protein Data Bank repository (https://www.wwpdb.org/) available from Alphafold (https://alphafold.ebi.ac.uk/) and elaborated using RasTop (https://www.geneinfinity.org/rastop/), as previously done in Pettersson and Cattaneo [39]. For IR75d, we used the PDB of *D. melanogaster* DmelIR75d (Q9 VVU7) highlighting the conserved arginine residue assigned for carboxyl group binding [12, 42] and non-conserved ligand binding domain (LBD) residues resulting from our polypeptide sequence alignment. Polypeptide sequence alignments were performed using Muscle [43] and manually refined with BioEdit [44]. Transmembrane domains were predicted with TopCons [45], S1/S2 subunits and pore loops have been assigned according to characterizations from Prieto-Godino et al. [21] and Benton et al. [8]. To analyze CpomIR41a1 we used the same methods to align its polypeptide sequence with other IR41a-candidates deposited in Alphafold, additionally adding *D. melanogaster* IR25a (GenBank: NP_001260049.1) and IR76b (GenBank: NP_649176.1) (Supplementary Figure S2, Supplementary Table S3). When adjusted by BioEdit, the alignment was adapted based on findings from Benton et al. [8]. To perform structural analysis of CpomIR41a1 we used the PDB of *Heliconius melpomene rosina* (A0 A140G9G8), whose polypeptide sequence resulted to be the most identical and similar to CpomIR41a1 among the IR41a sequences with an available PDB-structure (similarity matrix: BLOSUM62). IR41a-structural domains were assigned based on Benton et al. [8] and based on results from our polypeptide sequence alignment (Supplementary Figure S2). For comparative reasons, in support to the structural analysis of CpomIR41a1, we conducted further structural analysis by elaborating PDB-accessions of CpomIR25a (H9 A5R7) and CpomIR76b (H9 A5S5). To conduct structural analysis of IR64a, we analyzed the PDB-accession from the *D. melanogaster* orthologue (Q9 VRL4).

### Cloning and heterologous expression in human embryonic kidney cells

The complete ORFs of *C. pomonella* CpomIR41a1, CpomIR25a, CpmIR76b and *D. suzukii* DsuzIR8a cloned in pcDNA3.1 plasmids were synthesized by GeneArt™ gene synthesis service (Thermo Fisher Scientific, Waltham, MA, USA) adding a 5’-upstream CACC Kozak-sequence, as done previously [46] and employing codon optimization for the expression in human cells [47]. *D. melanogaster* IR8a and IR84a cloned into pcDNA3.1 were courtesy provided by Dr. Hayden R. Schmidt (International AIDS Vaccine Initiative, USA). Human Embryonic Kidney (HEK293T) cells were grown to semi-confluence in 35-mm Petri dishes containing HEK cell media [Dulbecco’s modified Eagle’s medium containing 10% fetal bovine serum (MP Biomedicals, Solon, OH, United States), 2 mM L-glutamine, and 100 mg/mL penicillin/streptomycin (Invitrogen)] at 37 ºC and 5% CO_2_. For CpomIR41a1-experiments, transient expression was conducted co-transfecting 2.0 μg of pcDNA5/TO-CpomOrco (obtained in Cattaneo et al. [46]) with same amounts of pcDNA3.1-CpomIR25a, pcDNA3.1-CpomIR76b and pcDNA3.1-CpomIR41a1. To report expression, 1.0 μg of pEBFP2-Nuc (Addgene #14893) [48], which contains the ORF of a blue fluorescent protein (EBFP), was also co-transfected. To monitor calcium, 1.0 μg of a separate plasmid carrying the ORF of a fluorescent calcium reporter, previously prepared in our lab in the frame of a different project (pEZT-BH-GCaMP, Dr. Hayden R. Schmidt) was co-transfected in substitution of pEBFP2-Nuc. To monitor voltage, 1.0 μg of a separate plasmid carrying the ORF of the voltage indicator ArcLight-Q239 (AddGene #36856) [49] was co-transfected in substitution of pEBFP2-Nuc. Expression of all reporter genes and chemosensory genes was under the regulation of the same CMV promoter. Co- transfections were achieved by mixing plasmids with 3.0 μL of FUGENE (Fugent LLC, Middleton, WI-USA) per μg of DNA and incubating cells overnight for up to 48 h. After incubation, HEK cell media was replaced with 2.0 mL fresh media and cells were incubated at 37 ºC for up to 6–8 additional hours. Part of the cell culture was then split in the middle of a MatTek P35G-1.5–14-C dishes (Ashland, MA USA) to obtain individual cells or small cell clusters, and rinsed at the sides with 2 mL fresh HEK media. After splitting, cells were allowed to recover for at least 1 day prior to imaging. Immunohistochemistry to confirm DsuzIR8a expression was performed following protocol described in Cattaneo et al. [46], combining a Guinea pig polyclonal Anti-DmelIR8a primary antibody with an Alexa488 anti-Guinea pig goat-polyclonal (Jackson ImmunoResearch, USA; http://www.jacksonimmuno.com). In a control experiment to test the expression of the DmelIR8a subunit, HEK-cells were transfected with 2.6 μg of pcDNA3.1 carrying the coding sequence of DmelIR8a (Dr. Hayden R. Schmidt).

### Imaging experiments

The activation of transfected HEK293T cells was assessed following the previously reported protocol [46]. Cell media of MatTek petri dishes was replaced with 2 mL of HEK Ca^++^ Ringer buffer (mM: 140 NaCl, 5 KCl, 2 CaCl_2_, 10 HEPES, pH 7.4). To test HEK293T cells for sodium (Na^+^) or potassium (K^+^) permeability, MatTek petri dishes were incubated for 1 h at room temperature in 1 mL HEK Ca^++^ Ringer, containing either the fluorescent indicator for Na^+^ or for K^+^ (NaTRIUM Green-2 AM and ION Potassium Green-4 AM respectively, ION Indicators, San Marcos, TX USA), both prepared at 5–15 mM with 0.06–0.2% Pluronic F-127 (Invitrogen). The buffer was removed after incubation, cells were rinsed with 4 mL fresh HEK Ca^++^ Ringer and placed on the stage of a Zeiss confocal microscope LSM710 using a 20x objective. Settings were adjusted based on single preparations, visualizing cells using 488-laser at 4%, gain 700–900. Cells were continuously perfused with Ca^++^ Ringer in the course of the experiment using a home-made gravity fed perfusion system. The perfusion system was constructed by combining two syringes (test VS wash) on a solid tube supporter with connected silicon tubes, and adjusting the height of the syringes to a chronometric flow-rate proximal to 400 μL/min. Silicon tubes terminated in a hand-valve, regulating wash after the stimulus at approximately 65% of the experiment. To provide stimulus on transfected cells, one single silicon tube was directed from this valve to an iron saw terminating with a plastic ClipTip pipette tip (Fisher Scientific), which was placed in the center of the preparation. Extra buffer was gently removed from the MatTek petri dish by the use of an additional silicon tube connected to an Ismatek IP-4 peristaltic pump (Fisher Scientific). Fluorescence imaging was performed in time-lapses setting the time-series of the microscope at 100–140 cycles of 1.0 s. After recording, fluorescent analysis was performed by ImageJ assigning to enough green-fluorescent cells a specific region of interest (ROI), for which changes in fluorescence intensity were measured by the tool “ROI manager”. Average fluorescence of the background was subtracted from the average fluorescence of each cell at each single time series. For each cell, the baseline fluorescence at the first capture (time 0) was subtracted from the average fluorescence at each single time series. Changes in fluorescence intensity were expressed as the fractional change in fluorescence intensity (∆F).

## Results

### Functional characterization of *C. pomonella* IR41a1 expressed in *D. melanogaster* olfactory sensory neuron alongside a native IR

We expressed *C. pomonella IR41a1* (*CpomIR41a1*) in the ac4 sensillum of *D. melanogaster* using an *IR76a-Gal4* driver line [17] (Fig. 1B). Consequently, CpomIR41a1 was co-expressed in the same OSN along with the native IR76a tuning subunit (DmelIR76a), and the co-receptors DmelIR25a and DmelIR76b. Following this, we screened the responses of transgenic and control ac4 sensilla to a panel of 23 compounds (Table 1), which included compounds known to activate several IR-expressing OSNs in *D. melanogaster* (housed in sensilla ac1, ac2, ac3 and ac4) as well as previously untested compounds. Among these latter, we observed consistent spiking activity in response to hexylamine and butylamine in both control and transgenic ac4 sensilla. This observation was consistently documented in all the control lines tested (Fig. 1) indicating that these two compounds may serve as previously undescribed agonists for the ac4 sensillum in *D. melanogaster*.

CpomIR41a1 expression conferred robust responses to pyridine (*p* = 0.005), putrescine (*p* < 0.001), cadaverine (*p* = 0.027) and spermidine (*p* < 0.001) compared to neurons from wild-type Oregon flies (Fig. 1A, B, Supplementary Data File 1, Supplementary Table S2), indicating that these four ligands are agonists of CpomIR41a1. Additionally, a slight inhibition of spiking activity in ac4 sensilla was observed with β-citronellol (*p* = 0.02), indicating a potential role of this volatile compound as antagonist of CpomIR41a1. Interestingly, the expression of CpomIR41a1 abolished trimethylamine inhibition (Oregon, *p* = 0.043; transgenic, *p* = 1), as well as evoked responses to stimuli known to activate DmelIR84a-expressing OSNs, like dimethylamine (*p* = 0.89), phenylacetaldehyde (*p* = 0.12) and phenylacetic acid (*p* = 0.17). This implies that the expression of CpomIR41a1 may interfere with the proper response of other OSNs housed in the same sensillum. However, spiking responses of the transgenic ac4 sensilla to pyrrolidine, which is a well-documented ligand of the native DmelIR76a [10, 17] co-expressed with transgenic CpomIR41a1, were quantitatively similar to those of the control ac4 sensilla (Fig. 1A, B). Conversely, response to 2-phenylethylamine, which is another DmelIR76a-ligand, presented with a lower average magnitude (19.16 ± 6.73 Δspikes/0.5 s) when compared with the effect on the wild type sensilla (40.17 ± 9.68 Δspikes/0.5 s). Indeed, by co-expressing CpomIR41a1 alongside the native DmelIR76a, we do not discard the possibility that the heterologous IR may form a complex with the endogenous IR-subunits, which may, most likely, differ in terms of the response to the common IR76a-ligands. However, the response to this ligand in CpomIR41a1-expressing neurons maintains statistical significance when compared with the solvent (*p* = 0.017, Supplementary Data File 1), and despite that the average magnitude of the response was different than that of the wild-type sensilla, statistical analysis unveiled absence of significant differences comparing data from the various replicates (Two-samples T-test, *p* = 0.1809; Mann Whitney U-test, *p* = 0.3176, Supplementary Data File 1). This suggests that expression of a second IR in the same OSN was most likely not interfering with the activity of the native receptor.

### Functional characterization of *D. suzukii* IR75d expressed in IR25a-based “ionotropic receptor decoder” of *D. melanogaster*

The *D. suzukii DsuzIR75d* gene was expressed in the OSN of a transgenic *D. melanogaster* that was lacking the expression of its own *DmelIR75d* but expressed the co-receptor *DmelIR25a*. DmelIR75d is known for being activated by pyrrolidine, as is DmelIR76a [17]. Consequently, SSRs from ac4 sensilla always showed a response to this ligand, even in transgenic lines that did not express DmelIR75d (Fig. 1C). However, when DsuzIR75d was expressed in *D. melanogaster* ac4 sensilla, they became sensitive to stimulation to hexanoic acid (*p* = 0.02). In turn, ac4 sensilla from the line lacking DmelIR75d or the wild type were not activated by hexanoic acid (Fig. 1, Supplementary Data File 1). Additionally, the expression of DsuzIR75d reduced the firing frequency of ac4 sensilla when exposed to triethylamine (*p* = 0.011), indicating a potential role of this volatile as antagonist of also DsuzIR75d, as we observed for the wild type (Oregon, *p* = 0.043). The heterologous expression of DsuzIR75d also abolished butylamine-evoked responses compared to control (*p* = 0.436), suggesting that the transgenic expression somewhat interferes with the neuronal response of ac4 sensilla.

### Functional characterization of *D. suzukii* IR64a expressed in IR8a-based “ionotropic receptor decoder” of *D. melanogaster*

To deorphanize the IR64a tuning subunit from *D. suzukii* (DsuzIR64a), we employed a “ionotropic receptor decoder'' approach similar to that used for DsuzIR75d and already used in the deorphanization of *D. melanogaster* and *D. sechellia* IR subunits [10, 14, 20–22]. In this case, we drove the expression of DsuzIR64a in the OSN that lacks the expression of native DmelIR84a but still expresses the co-receptor DmelIR8a (Fig. 1F). This choice was based on the fact that in *D. melanogaster*, the DsuzIR64a orthologue, DmelIR64a, is dependent on the presence of the co-receptor DmelIR8a [13, 19]. Hence, we hypothesized that the conserved orthologue from *D. suzukii* would also require an IR8a co-receptor for proper functioning.

The results showed that there were no novel odor-evoked responses in mutant ac4 sensilla expressing DsuzIR64a compared to control ac4 sensilla lacking DmelIR84a (Fig. 1E, F). Specifically, we did not observe any response to the ligands known to activate the orthologue DmelIR64a, which senses carboxylic acids [13, 19]. This suggests that under our experimental conditions, the expressed IR tuning subunit was either non-functional or had a shift in its binding affinity towards ligands not included in the tested panel. However, the expression of DsuzIR64a interfered with the normal firing activity of ac4 sensilla, reducing their spike frequencies when exposed to pyrrolidine (*p* = 0.174), dimethylamine (*p* = 0.462) and ammonium hydroxide (*p* = 0.175) compared to the solvent, while the control ac4 sensilla lacking DmelIR84a maintained a significant effect (Fig. 1F; Supplementary Data File 1). Interestingly, a reduction in firing activity of ac4 was observed also for hexylamine (27.2 ± 5.46 Δspikes/sec) when compared with the control ac4 sensilla lacking DmelIR84a (39.83 ± 9.69 Δspikes/sec) despite that its effect remained significant when compared with the solvent (Two-sample T-test, *p* = 0.005). In a parallel set of experiments, we expressed in ac4 sensilla lacking DmelIR84a a truncated isoform of the *C. pomonella* orthologue CpomIR64a, which lacks the transmembrane region M3 and is non-functional (CpomIR64a∆). We again observed a similar reduction in firing activity of mutant ac4 sensilla in response to some of the aforementioned stimuli (pyrrolidine, *p* = 0.276; dimethylamine, *p* = 0.816; ammonium hydroxide, *p* = 0.058) (Fig. 1G). This suggests that heterologous expression of novel IR subunits, even if non-functional, may interfere with the native neuronal activity of other OSNs housed in the same sensilla.

In addition, we observed a significant response to ethanol when both transgenic fly lines, DsuzIR64a and CpomIR64a∆, were compared with the wild-type Oregon line (Two-sample T-test, *p* = 0.0208 and *p* = 0.0345 respectively). This solvent effect was not observed when Oregon was compared to the empty decoder neuron flies lacking DmelIR84a (Two-sample T-test, *p* = 0.5463). Therefore, the effects of compounds diluted in ethanol must be interpreted with caution. Among these compounds, only 2-phenylethylamine elicited a clear response in the ac4-sensilla from the IR64a-transgenic flies tested. Spiking in response to this ligand was evident (21.00 ± 6.89 and 26.33 ± 6.63 spikes/0.5 s for DsuzIR64a and CpomIR64a∆, respectively), especially when compared with ethanol alone (3.60 ± 1.29 spikes/0.5 s and 5.00 ± 2.46 spikes/0.5 s). Furthermore, for both IR64-transgenic lines, the response to ethanol was not significantly different from the response to water (Paired T-test: *p* = 0.39 and *p* = 0.47 for DsuzIR64a and CpomIR64a∆, respectively Supplementary Data File 1), suggesting this ethanol is unlikely to act as an active ligand. Nonetheless, the limited influence of the solvent on ligand-enhanced spiking cannot be excluded.

### Dose–response characteristics of CpomIR41a1 and DsuzIR75d

**Fig. 2:**
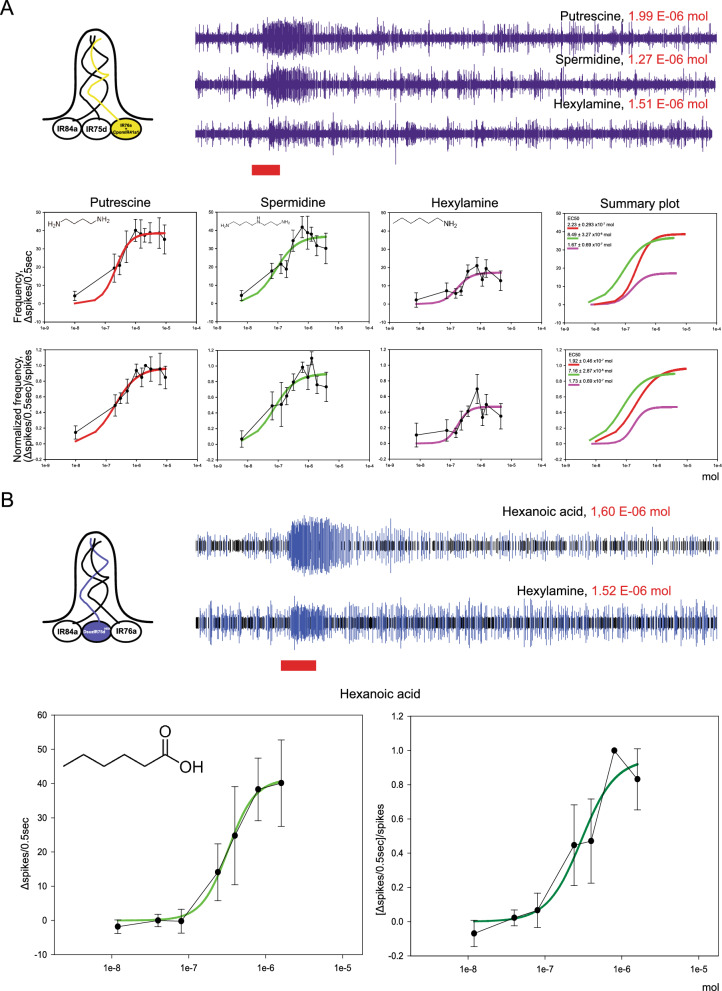
Dose–response characteristics of ac4 sensilla from transgenic lines expressing *CpomIR41a1* and *DsuzIR75d*^*HEK*^. (**A**) Dose–response to putrescine, spermidine and hexylamine of ac4 sensilla from antennae of *w;IR76a-Gal4;UAS-CpomIR41a1* fly lines. Above left: schematic representation of ac4 sensilla from transgenic *D. melanogaster* co-expressing CpomIR41a1 in IR76a-neurons as in Fig. 1B; right: representative traces of spike from ac4 sensilla generated by DOSE 20 of the specific stimuli (putrescine: 1.99 E-06 mol; spermidine: 1.27 E-06 mol; hexylamine: 1.51 E-06 mol). The red box below the traces marks the stimulus time. Below, upper panels: data expressed as a function of spike frequency (Δspikes/0.5 s); lower panels: data expressed as normalized frequency [(Δspikes/0.5 s)/spikes]. Spike frequencies were normalized to the effect of saturating doses of putrescine (DOSE 20: 1.99 E-06 mol), these plots resulted with similar trends from the ones before normalization. Data are presented as mean ± SEM and fit with the Hill equation (solid lines). The rightmost graphs represent the summary plots (putrescine, red; spermidine, green; hexylamine, magenta); top left: EC50s, as in Table 2. (**B**) Dose–response to hexanoic acid of ac4 sensilla from antennae of *w;IR75d-Gal4/UAS-DsuzIR75d*^*HEK*^*;IR75d*^*KO*^ fly lines. Left: schematic representation of ac4 sensilla from transgenic *D. melanogaster* that expressed DsuzIR75d^HEK^ instead of DmelIR75d in its specific neurons as in Fig. 1D; right: representative traces of spike from ac4 generated by DOSE 20 of hexanoic acid and DOSE 20 of hexylamine. The red box below the traces marks the stimulus time. Below, left panel: data expressed as a function of spike frequency (Δspikes/0.5 s); right panel: data expressed as normalized frequency [(Δspikes/0.5 s)/spikes]. Spike frequencies were normalized on the effect to saturating doses of putrescine (DOSE 10 to 20: [8, 16] E-07 mol). Data are presented as mean ± SEM and fit with the Hill equation (solid lines)

We compared the odor responses to amines conferred by CpomIR41a1 expression by generating dose–response curves, as well as responses to the newly described ac4 activator, hexylamine. Mutant ac4 sensilla expressing CpomIR41a1 responded to putrescine, spermidine and hexylamine in a dose-dependent manner (Fig. 2A, Supplementary Data File 1, Supplementary Data File 2) and the pharmacological parameters are indicated in Table 2. Results suggest that CpomIR41a1 is more sensitive to spermidine than putrescine (Fig. 2A), as reflected by the EC50 values. Overall, our findings provide a successful example of heterologous IR deorphanization using co-expression of a transgenic IR alongside a native IR.

**Table 2:** pharmacological parameters of transgenic ac4 sensilla expressing CpomIR41a1 and DsuzIR75d

Normalization	Parameters	ac4 expressing CpomIR41a1			ac4 expressing DsuzIR75d
		Putrescine	Spermidine	Hexylamine	Hexanoic acid
Not normalized	EC50 (mol)	2.23 ± 0.293 × 10^–7^	8.49 ± 3.27 × 10^–8^	1.67 ± 0.69 × 10^–7^	3.26 ± 0.196 × 10^–7^
	Hill coeff	1.833 ± 0.611	1.24 ± 0.7201	1.864 ± 1.51	2.501 ± 0.39
	Fmax^a^(spks^b^/0.5 s)	38.62 ± 1.59	36.84 ± 4.12	17.21 ± 2.85	41.38 ± 1.7
Normalized	EC50 (mol)	1.92 ± 0.46 × 10^–7^	7.16 ± 2.87 × 10^–8^	1.73 ± 0.69 × 10^–7^	2.98 ± 0.89 × 10^–7^
	Hill coeff	1.15 ± 0.44	1.2 ± 0.68	2.31 ± 2.17	1.97 ± 1.11
	Fmax^a^[(spks^b^/0.5 s)/spks^b^]	0.97 ± 0.07	0.90 ± 0.094	0.47 ± 0.08	0.95 ± 0.17
Saturation (mol)		1.99 × 10^–6^	6.37 × 10^–7^	7.57 × 10^–7^	[0.80; 1.60] × 10^–6^

^a^Maximal effect

^b^Spikes

Further characterization showed that mutant ac4 sensilla expressing DsuzIR75d responded to hexanoic acid in a dose-dependent manner (Fig. 2B) supporting that this is an agonist of DsuzIR75d. The inhibitory effect we observed for triethylamine and the abolished response to butylamine (Fig. 1D) were also tested in dose response but, although the increment of doses showed constant inhibition, we did not observe dose–response characteristics (Supplementary Figure S3). Overall, these results demonstrate that the use of a novel “ionotropic receptor decoder” based on a neuron expressing the co-receptor DmelIR25a but lacking its native receptor is suitable for IR characterization from non-model species.

### Structural analysis of CpomIR41a1

**Fig. 3:**
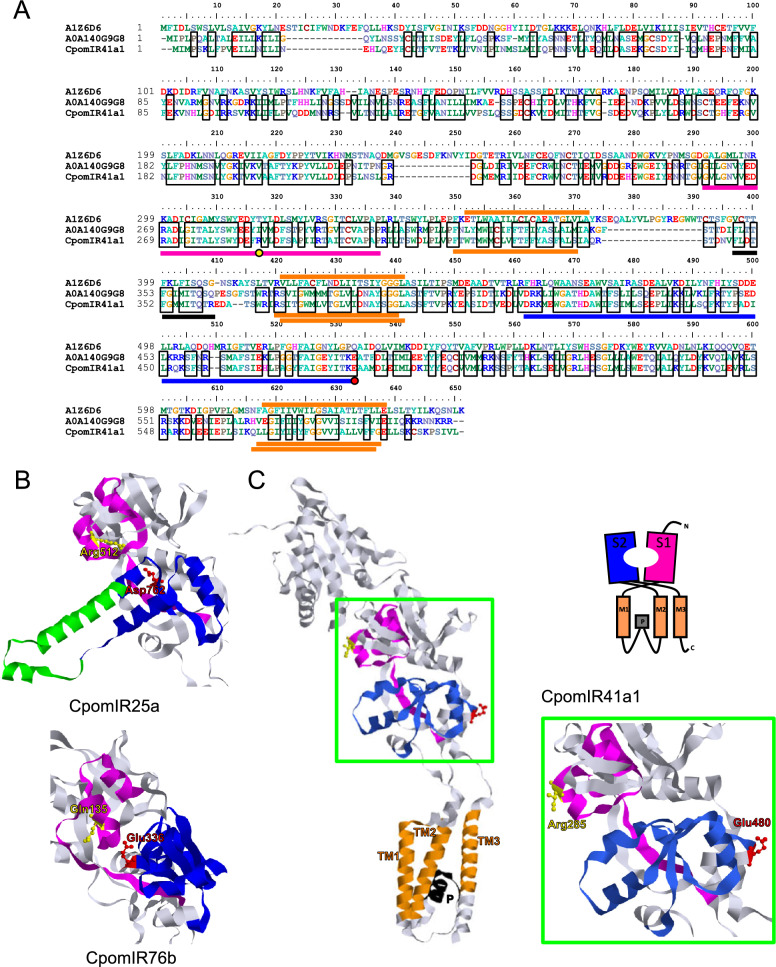
Structural analysis of CpomIR41a1. (**A**) Polypeptide sequence alignment of CpomIR41a1 with the orthologues from *H. melpomene rosina* (PDB: A0 A140G9G8) and *D. melanogaster* (PDB: A1Z6D6). Black squares depict identical sequence between *C. pomonella* and *H. melpomene rosina*; bars indicate structural domains as S1 (magenta), pore loop (black), S2 (blue) and transmembranes (orange: above, *D. melanogaster*; below, *H. melpomene rosina* and *C. pomonella*). Red dot: Glutamate residue at the end of S2 as indicated in Benton et al. [8]. (**B**) Structural analysis of the LBD of the co-receptor subunits: CpomIR25a (above) and CpomIR76b (below). the green ribbon in CpomIR25a highlights the S2-CREL according with Abuin et al. [9]. (**C**) Structural analysis of the CpomIR41a1 subunit (left), highlighting all functional domains represented in the scheme on the top right, and the magnification of the LBD (right). Colors highlighting domains and S1/S2 key residues are used as in A

The polypeptide sequence alignment between CpomIR41a, and other orthologues deposited in PDB (Supplementary Figure S2) unveiled *H. melpomene rosina* having the highest sequence identity (~ 0.56) and similarity of (~ 0.78). When aligned together with the orthologue from *D. melanogaster*, which has been up to now functionally characterized, the alignment presents few gaps, which are mostly located within the N-terminus and in proximity of the pore loop (Fig. 3A). Among these, three main gaps ranging from 13 to 16 amino acids resulted from the presence of extra sequence residues from the *D. melanogaster* subunit at positions 22–29, 238–250 and 374–389. Interestingly, polypeptide sequence comparison resulted in short S1 (43 residues) and S2 (69 residues) domains. Comparing the positioning of transmembrane domains, TopCons unveiled conserved positions for TM1, and 1-residue shifts for TM2 and TM3 between the two lepidopterans. Instead, the orthologue of *D. melanogaster* presents TM1 and TM3 shifts of 2 residues forward when compared with the CpomIR41a1-orthologue. Interestingly, the polypeptide sequence alignment (Supplementary Figure S2) demonstrated the presence of an arginine residue within the S1 subunit of CpomIR41a1 (Arg285), which is known in iGluRs for binding to the α-carboxyl group of the glutamate ligand. In addition, at the end of the S2-subunit of CpomIR41a1, the alignment demonstrated the conservation of a negatively charged residue between *C. pomonella* (Glu480) and *H. melpomene rosina* (Glu483), renowned in iGluRs for participating in binding of the α-amino group from the glutamate ligand [4]. Comparing co-receptors (Fig. 3B), the S1-arginine is conserved also for CpomIR25a (Arg512), but it is substituted by glutamine in CpomIR76b (Gln135). Instead, the S2-negatively-charged residue is present for both CpomIR25a as an aspartate (Asp762), and for IR76b as a glutamate (Glu336), as is the case for CpomIR41a1. Comparing structural analysis among Cpom-co-receptors and CpomIR41a1 (Fig. 3B,C) we observed that while for the co-receptors the lateral chain of these residues protrude towards the inside of the LBD (Fig. 3B), for the CpomIR41a1 subunit they instead protrude externally (Fig. 3C). Finally, our polypeptide sequence alignment demonstrated that both the S1-arginine and the S2-negatively charged residues are conserved also for other IR41a-subunits (Supplementary Figure S2, Supplementary Table S3) despite that they were not identified in the IR41a subunit of *D. melanogaster*.

### Structural analysis of DsuzIR75d

**Fig. 4:**
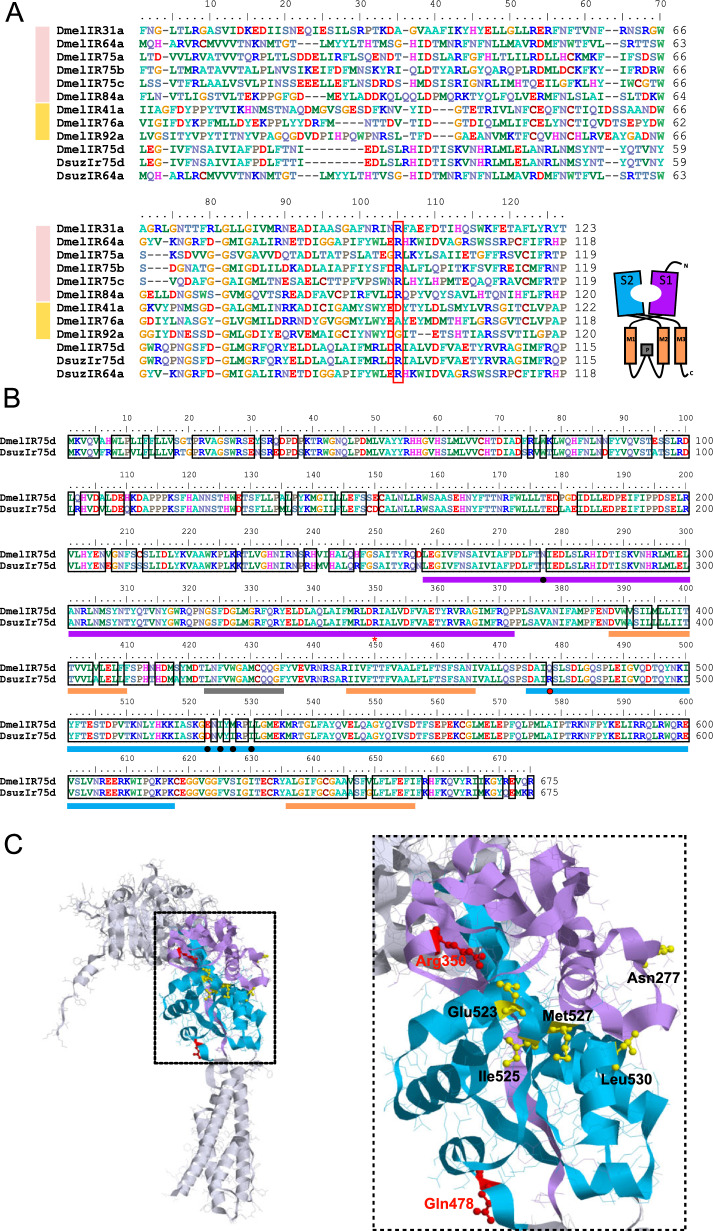
Sequence comparison and structural analysis of IR75d orthologs. (**A**) Sequence alignment of the S1 region of DsuzIR75d, DmelIR75d and DsuzIR64a with all *D. melanogaster* IRs known to bind acids (pink bar) and amines (yellow bar). Red square: arginine proximal to the end of the LBD-S1 domain which is conserved in iGluRs and acid-sensing IRs, assigned for carboxyl group binding [12, 42]. (**B**) Alignment of *D. suzukii* and *D. melanogaster* IR75d orthologues. Lilac filled squares indicate the S1 lobe, light-blue filled squares indicate the S2 lobe of the predicted ligand-binding domain (LBD), based on Prieto-Godino et al. [21], represented in the scheme on the top right. Orange bars indicate the transmembrane regions, and the gray bar illustrates the re-entrant pore loop. Black boxes in the alignment highlight amino acids conserved between *D. suzukii* and *D. melanogaster*. Asterisk: conserved Arg350 [12, 32]; bullets: amino acid substitutions in S1/S2 lobes: black, conserved substitutions; red: non-conserved. (**C**) Protein model based on DmelIR75d (PDB: Q9 VVU7) generated by RasTop (left) and magnified view of the ligand-binding domain (right). Lilac ribbons: S1-LBD; light-blue ribbons: S2-LBD. Red residues: positions of the conserved Arg350 [12, 42] and of the Gln/Arg478 residue that is indicated by a red bullet in B. Yellow residues: conserved amino acid substitutions within the S1/S2 between the *D. melanogaster* and *D. suzukii* orthologues, as indicated in B: Asn/Thr277, Glu/Asp523, Ile/Val525, Met/Ile527 and Leu/Ile529. Note proximity of the positions 350, 523, 525, 527 and 529 in the S1/S2 pocket of the LBD

To support the role of DsuzIR75d as an acid-sensing IR, we aligned the sequence of the region S1 of the ligand binding domain (LBD) with those of *D. melanogaster* IRs*.* This was done because it has been observed that a conserved arginine residue exists in all acid-sensing IRs (DmelIR31a, DmelIR64a, DmelIR75a, DmelIR75b, DmelIR75c, and DmelIR84a), while it diverges in amine-sensing IRs (DmelIR41a, DmelIR76a, and DmelIR92a) [10, 12]. This residue is thought to play a crucial role in binding the carboxyl group (C(= O)OH) of ligands. We discovered that this residue is also conserved in DsuzIR75d (Fig. 4A), which we demonstrated to be activated by hexanoic acid. However, it is also present in DmelIR75d, which does not respond to carboxylic acids. When we further compared the sequences of DmelIR75d and DsuzIR75d, we identified 56 divergent amino acids across the alignment. Among these, one was located within the S1 segment of the LBD (Asn/Thr277), and five were found in the S2 segment (Glu/Asp523, Ile/Val525, Met/Ile527, Leu/Ile529 and Gln/Arg478, Fig. 4B). Structural analysis of IR75d unveiled four out of these six LBD substitutions (Glu/Asp523, Ile/Val525, Met/Ile527, Leu/Ile529) positioned in proximity of the S1/S2 binding pocket (Fig. 4C). Among these six substitutions, only Gln/Arg478 was non-conserved since it led to the change from a non-polar to a positively charged amino acid. The alignment of all Dmel IRs deorphanized so far unveiled that a positively charged residue is present in that position in all acid sensing IRs, except for IR31a (Supplementary Figure S4). Additionally, we observed four differences in transmembrane region 1 (TM1) and two in transmembrane region 3 (TM3). TM2 and the re-entrant pore loop were identical to those of *D. melanogaster*.

### Structural analysis of DsuzIR64a

**Fig. 5:**
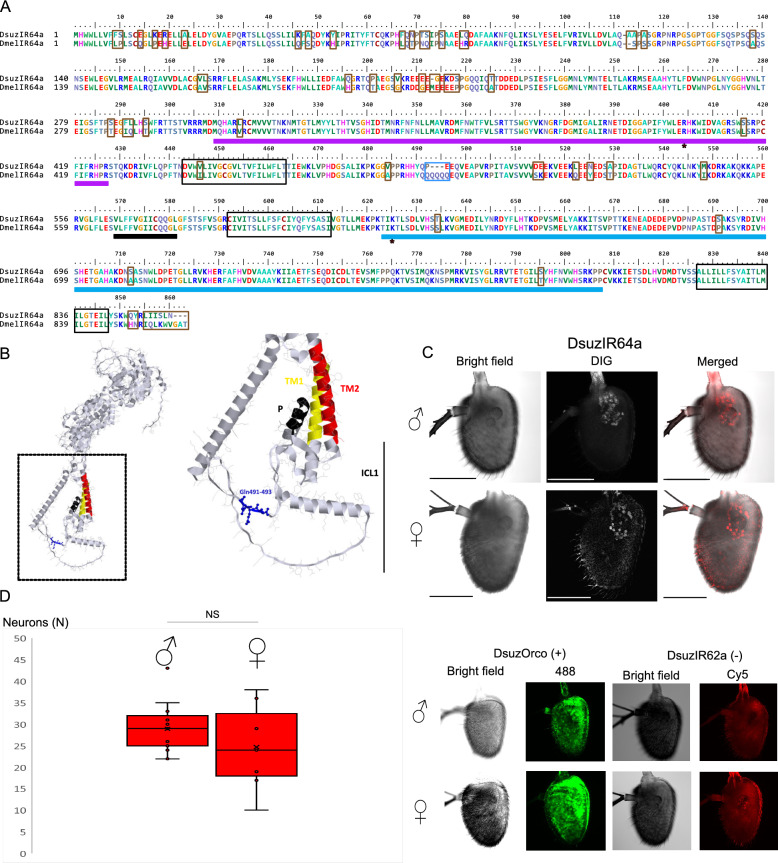
Structural analysis and fluorescent in situ hybridization of DsuzIR64a. (**A**) Polypeptide sequence alignment of IR64a orthologues from *D. suzukii* and *D. melanogaster*. Amino acid substitutions, insertions and deletion between *D. suzukii* and *D. melanogaster* are represented as brown opened squares, while the deletion of the glutamine residues is represented as a blue opened square. Structural domains are represented as filled squares (LBD, S1; lilac; S2: light blue; pore loop: black. Transmembrane domains are indicated as opened black squares. Red asterisks depict positions of Arg402 [12, 42] and of the Lys623 residue, which is conserved in all acid sensing IRs, except for IR31a (Supplementary Figure S4). (**B**) Left: protein model of DmelIR64a generated by RasTop. Right: magnified view of the ICL-1 of DmelIR64a. Blue: glutamine residues absent in the subunit of *D. suzukii*. Black: pore loop; yellow: Transmembrane domain M1; red: transmembrane domain M2, as described in Benton et al. [8]. (**C**) Fluorescent in situ hybridization of the *DsuzIR64a* RNA of a male (♂) and a female (♀) antenna. From left to right: bright field, Cy5 and merged channels. Below: parallel control experiments have been conducted with specific probes against *DsuzOrco* RNA (positive control) and *DsuzIR62a* RNA (negative control). Scale bar: 100 μm. (**D**) Boxplots illustrate counting of neurons from C labeled by anti-*DsuzIR64a* RNA probe in males and females (N_male_ = 15, N_female_ = 7). NS: no significant differences (Two-samples T-test, *p* = 0.339)

Since DsuzIR64a did not respond to the carboxylic acids known to activate the orthologous DmelIR64a (Fig. 1F), we carried out a sequence alignment of *D. melanogaster* and *D. suzukii* IR64a (Fig. 5A) to identify the likely molecular basis of the lack of response to known DmelIR64a ligands. Structural analysis (Fig. 5B) was based on the *D. melanogaster* orthologue (PDB accession: Q9 VRL4), in which we highlighted features relevant to the structure and function of this protein. In a supplementary analysis (Supplementary Figure S5), we investigated LBD highlighting the amino acids that we have identified substituted between *D. melanogaster* and *D. suzukii* (Fig. 5A).

This analysis revealed a high degree of conservation across the two drosophilid species. However, in DsuzIR64a, we observed fifty-eight amino acid substitutions, one small insertion and three small deletions, when compared to *D. melanogaster* orthologue. These differences were mainly located in the N-terminal part of DsuzIR64a, which is displayed externally on the cell surface. In contrast, the LBD of DsuzIR64a, crucial for receptor targeting and chemical recognition, differed from those of *D. melanogaster* by only six amino acid substitutions (two in S1 and four in S2 subunits) (Fig. 5D). The arginine residue conserved among acid-sensing IR is also conserved in DsuzIR64a (Fig. 4A, Supplementary Figure S4). A further conserved substitution was detected in transmembrane segment 1 (TM1) while the sequences of TM2, TM3 and the re-entrant pore loop between TM1 and TM2 were identical to those of *D. melanogaster*. TM1, TM2 and the re-entrant pore loop collectively form the ion channel pore, which is the most conserved region between IRs and iGluRs and controls ion conductance [12, 42]. While TM1, TM2 and the pore loop were virtually identical among the species, we detected three amino acid deletions in the intracellular loop 1 (Fig. 5B), located between TM1 and the pore loop, specifically in *D. suzukii*. Here, three consecutive glutamines were missing in the DsuzIR64a subunit compared to *D. melanogaster*.

Our choice to express DsuzIR64a in the IR8a-based “ionotropic receptor decoder” of *D. melanogaster* (Fig. 1F) was based on the hypothesis that a conserved orthologues from *D. suzukii* (Fig. 5A) would also require an IR8a co-receptor for proper functioning. This hypothesis was further supported by the expression pattern of DsuzIR64a, which closely resembles previous observations in *D. melanogaster* for DmelIR64a [8, 13]. Indeed, FISH experiments showed that *DsuzIR64a* is expressed in ~ 26 OSNs located near the sacculus (Fig. 5C). Furthermore, there are no significant differences between *D. suzukii* males and females (Fig. 5D) (Two samples T-test: *p* = 0.339, Supplementary Data File 3, Supplementary Table S2).

### Heterologous expression of insect IRs in HEK293T cells

**Fig. 6:**
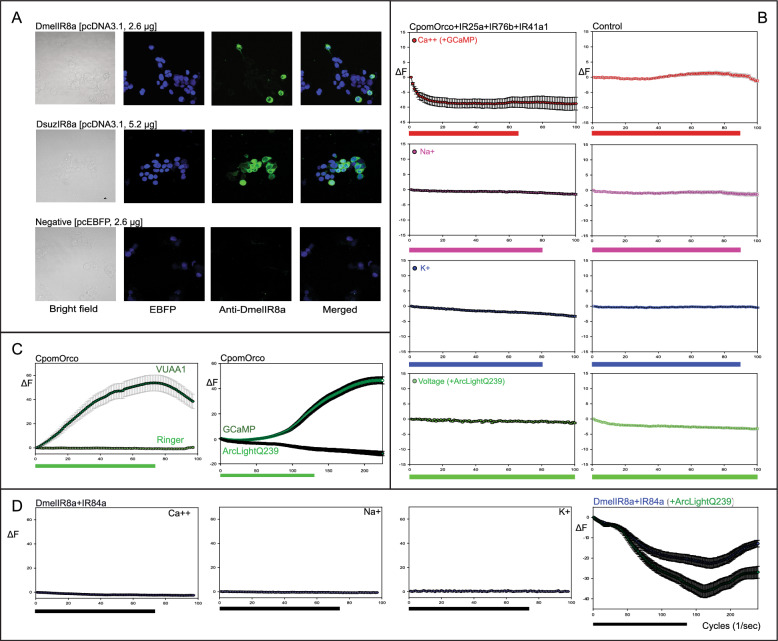
Heterologous expression of IR subunits in HEK293T cells. (**A**) Immunohistochemistry of HEK293T cells expressing DsuzIR8a comparing a positive control expressing DmelIR8a (above), and a negative control (pEBFP, 2.6 μg), where cells were transfected with the sole plasmid carrying the coding sequence of the EBFP indicator (below). From left to right: bright field, EBPF expression (blue), anti-IR8a antibody (green), merged fields. (**B**) Variation of fluorescence measured from HEK293T cells co-transfected with CpomOrco + CpomIR25a + CpomIR76b + CpomIR41a1 (+ GCaMP to test fluorimetry dependent to Ca^++^ + ArcLightQ239 to test fluorimetry dependent to voltage) (left panels) and their respective controls (no transfection, GCaMP alone for Ca^++^, ArcLightQ239 alone for voltage, right panels) stimulated with pyridine 1–2 mM. Red dots show the time-course selectivity to Ca^++^ ions, magenta dots show selectivity to Na^+^, blue dots show selectivity to K^+^, and green dots show voltage responses. Stimulus duration (at least 65% of the experiment) varies according to the experiment (as represented by the color-coded bars below). Note: to monitor N^+^ and K^+^, Ca^++^-Ringer was enriched either with NaTRIUM Green-2 AM or with ION Potassium Green-4 AM, as described in the Methods section. (**C**) Positive control of VUAA1-enhanced fluorescence effects from CpomOrco comparing calcium- and voltage-dependent effects. HEK293T cells were co-transfected with CpomOrco + CpomIR25a + CpomIR76b + CpomIR41a1 subunits, together with the fluorescent GCaMP Ca^++^-indicator (left) or either the GCaMP indicator or the ArcLightQ239 voltage-indicator (right). The left panel shows Ca^++^ dependent fluorescent response to stimulation with 500 μM VUAA1 dissolved in DMSO in comparison with the ringer buffer lacking the ligand, but containing only DMSO (N = 53). Right: comparison of fluorescent response to stimulation with 500 μM VUAA1 monitoring Ca^++^ selectivity co-expressing GCaMP (N = 129) and the voltage-dependent fluorescence co-expressing ArcLightQ239 (N = 74). Decrement in fluorescence for ArcLightQ239 indicates access of cations into the plasma membrane [49]. Stimulus duration is represented by the green bars below. (**D**) Fluorescent measurement of HEK293T cells co-transfected with DmelIR8a + DmelIR84a, stimulated with 1.0 mM phenylacetic acid. Separated responses were displayed for Ca^++^ (+ GCaMP, N = 115) or Na^+^ (N = 89) and K^+^ (N = 54) (IR-subunits expressed alone). Bottom right: comparison of the fluorescent response of HEK293T cells co-transfected with DmelIR8a + DmelIR84a + ArcLightQ239 (blue, N = 67) with HEK293T cells transfected with the sole ArcLightQ239 (green, N = 59) and stimulated with 2.0 mM phenylacetic acid. Stimulus duration is represented by the black bars below denoting cycles as approximate units of time, based on settings from the confocal microscope (1 cycle/second). Dots represent the average ± SEM

We next explored the utility of an ex vivo system, specifically the HEK293T system, for heterologous expression and functional characterization of insect IRs. Initially, we optimized the expression protocol using *D. suzukii* DsuzIR8a. To confirm the correct expression of this transgene, we stained co-transfected HEK293T cells with an anti *D. melanogaster* IR8a conjugated with Alexa488 dye. This antibody should be capable of recognizing DsuzIR8a due to a high degree of sequence conservation of its antigen between Dmel/DsuzIR8a. As shown in Fig. 6A, the majority of co-transfected HEK293T cells carrying the pcDNA-3.1-DsuzIR8a showed the Alexa488 signal, suggesting a correct staining from the antibody. Furthermore, the signal was localized around the cytoplasm expressing the EBFP2 blue fluorescent protein (EBFP), which served as transfection control. This indicates proper expression of DsuzIR8a in the cell membrane, leading us to conclude that HEK293T cells are a suitable system for accurate IR expression.

Next, we co-transfected HEK293T cells with an expression plasmid carrying *CpomIR41a1*, alongside the broadly expressed co-receptors *C. pomonella* IR25a (CpomIR25a) and IR76b (CpomIR76b), which are required for its function. Additionally, a plasmid carrying the odorant receptor co-receptor Orco (CpomOrco) was also co-transfected as a positive control. We perfused the co-transfected cells with pyridine, a ligand we had previously demonstrated to activate CpomIR41a1 in in vivo expression experiments (Fig. 1B). We observed an overall decrement of fluorescence when testing cation permeability and voltage. Control experiments also revealed an apparent increment in fluorescence associated with calcium and a decrement in voltage-associated fluorescence, suggesting that the expression of the combination CpomIR41a-CpomIR25a-CpomIR76b in HEK293T cells did not produce a specific response (Fig. 6B). However, when we perfused VUAA1, an activator of CpomOrco [46], which was co-transfected with the plasmid carrying the three IRs, a clear response was observed when monitoring both calcium and voltage (Fig. 6C). This indicates that the system was functional and the lack of response from the IR combination was likely due to other effects.

Lastly, we attempted to use HEK293T cells to deorphanize a tuning IR which requires the IR8a co-receptor instead of IR25a. We specifically selected the *D. melanogaster* DmelIR84a since its main agonist phenylacetic acid is already known [14]. When we perfused phenylacetic acid we did not observe any discernible effects on cation sensitivity (Fig. 6D). However, the voltage indicator ArcLightQ239 unveiled a reduction in the amplitude of fluorescent variation, possibly indicating the DmelIR8a + DmelIR84a activation by phenylacetic acid. Nevertheless, we observed the same effect when ArcLightQ293 was expressed alone, suggesting that this decrease in fluorescence was likely an artifact. In conclusion, our results indicate that, under our experimental conditions, HEK293T cells are not suitable for the functional characterization of IRs.

## Discussion

While extensive research has delved into the understanding of IRs in *Drosophila* and a few selected mosquito species, there remains a significant knowledge gap regarding the role of these receptors in odor perception within other insect orders. This shortfall primarily stems from the limited availability of genetic tools in non-model species, which hampers the functional characterization of tuning IR subunits beyond *Drosophila*. In this study, we expanded the deorphanization strategy of the"ionotropic receptor decoder"from using an OSN that expresses the co-receptor DmelIR8a [20–22] to other OSNs expressing the co-receptor DmelIR25a or the combination of DmelIR25a and DmelIR76b. We demonstrated the efficacy of this strategy in characterizing IR tuning subunits not only from a fruit fly species outside the *D. melanogaster* subgroup but also from a distantly related order, Lepidoptera.

To achieve IR deorphanization, we exploited the other two OSNs housed in the ac4 sensillum together with the “ionotropic receptor decoder” used in previous study. After transgenic expression of heterologous IRs in ac4 sensillum, we used a panel of 23 volatile compounds that include chemicals known to activate IRs in *D. melanogaster* as well as previously untested volatiles. This drove us to the identification of a novel agonist for *D. melanogaster* ac4 sensillum: hexylamine. This compound was not included in the stimuli screened previously against this sensillum [8–10, 14, 17, 20–22, 50] The discovery of hexylamine as an agonist aligns with previous suggestions that sensing of hexylamine, which repels *D. melanogaster*, is mediated by OSNs other than those expressing DmelIR92a and housed in ac1 sensilla [15]. The next step will be to understand which IR tuning subunit expressed in ac4 sensillum (DmelIR75d, DmelIR76a and DmelIR84a) is responsible for hexylamine sensing. Our results showed that ac4 sensilla from both DmelIR84a and DmelIR75d knock-out lines exhibited a firing response to hexylamine similar to wild-type ac4 sensilla (Fig. 1A, C, E). This observation rules out the possibility that either of these two IR subunits is necessary for sensing hexylamine. However, it leaves open the possibility that both subunits may redundantly contribute to the response to this volatile compound. Further studies are needed to understand the contribution of these subunits and DmelIR76a in sensing hexylamine.

Our results showed a functional expression of *D. suzukii* and *C. pomonella* tuning IR subunits in *D. melanogaster* OSNs. Evolutionary studies have revealed that IR subunits diverge in terms of gene numbers and coding sequences across species, while the co-receptors remain highly conserved [3, 51]. This suggests that the assembly and function of heteromeric complexes are likely to be similar in different lineages. Indeed, transgenic expression in *D. melanogaster* DmelIR25a-expressing OSNs of a gene (*DsuzIR75d*) from *D. suzukii,* whose ancestor diverged from that of *D. melanogaster* around 15 million years ago [52] led to the formation of a functional heteromeric complex. The same happened for transgenic expression of the lepidopteran gene *CpomIR41a1*. This demonstrates the broad conservation of the IR-based olfactory system across insects and opens avenues for the widespread use of this technique in IR deorphanization. This approach may parallel the extensive use of the *Drosophila* “empty neuron system” for deorphanizing ORs from non-model species [29, 53, 54].

Additionally, the"ionotropic receptor decoder"system offers several advantages over the traditional ex vivo approach, such as heterologous expression in *Xenopus* oocytes, which has been used for deorphanization of a limited number of IRs from non-model species [23–27]. One of the main advantages of using an in vivo system like the "ionotropic receptor decoder" is that it closely mimics the native olfactory sensillum environment, ensuring a high-fidelity receptive field for the expressed receptor. Additionally, the vapor-phase odor delivery in the empty-neuron technique provides a more realistic physicochemical environment compared to water-phase odor delivery required by in vitro systems. Altogether, these differences contribute to the increased sensitivity of in vivo deorphanization compared to the oocyte expression system [55, 56]. However, there are also some disadvantages to using an in vivo system. The generation of transgenic fly lines and the subsequent electrophysiological recording experiments can be time-consuming and may not be suitable for high-throughput screenings (Supplementary Figure S6). In our experiments, we demonstrated that the deorphanization strategy based on transgenic expression of IRs in *D. melanogaster* OSNs can be achieved using both empty OSNs and OSNs that already express native IR subunits (Fig. 1, 2). This flexibility in expression methods facilitates the use of this technique, as it does not necessarily require the creation of transgenic *D. melanogaster* IR knockout lines. However, it is important to exercise caution and implement appropriate controls, as the firing profile of the native IR subunits may potentially mask the response of the transgenic IRs (Fig. 1).

The deorphanization of IRs beyond *Drosophila* enables comparative studies across families and orders. For instance, it allows testing if one-to-one orthologous IRs present in distant lineages maintain the same agonist specificity as observed in *D. melanogaster*. IR41a is one such example. It exhibits a one-to-one copy in all analyzed lepidopteran and dipteran genomes, albeit with additional copies found in some mosquito and lepidopteran species [3, 25, 51], including *C. pomonella*, where there are two putative paralogues [31]. In *D. melanogaster*, DmelIR41a forms a heteromeric complex with DmelIR25a and DmelIR76b, responding to polyamine ligands like pyridine, pyrrolidine, putrescine, cadaverine and spermidine [17, 18]. A similar activation pattern has been observed in the mosquito *A. gambiae* [25]. Our study extends this functional conservation beyond the Diptera order, as the mutant ac4 expressing CpomIR41a1 exhibited firing activity when stimulated with pyridine, putrescine, cadaverine, and spermidine (Fig. 1B). However, a major limitation in our characterization was the inability to test the response to pyrrolidine: another known IR41 agonist identified in *D. melanogaster* and *An. gambiae* [17, 18, 25]. Stimulation of CpomIR41a1 with pyrrolidine could not be evaluated due to spontaneous firing of ac4 sensilla caused by native DmelIR76a and DmelIR75d expression [17]. Additional experiments using IR76a/IR75d-knockout backgrounds, which could more specifically isolate CpomIR41a1 expression, may help clarify its potential response to pyrrolidine. Analyzing the polypeptide sequence alignment of IR41a variants (Supplementary Figure S2), we observed two residues from the venus-flytrap domains of CpomIR41a1 to be conserved with iGluRs: an arginine within the S1 domain (Arg285) and a negatively charged glutamate at the end of the S2 domain (Glu480). Both residues are known in iGluRs for participating in glutamate binding [8]: the positively charged arginine interacts with the α-carboxyl group of the ligand, while the negatively charged residue—either aspartate or glutamate—interacts with the α-amino group [4]. Notably, NMDAR iGluRs typically feature an aspartate at this site, while AMPAR iGluRs have a glutamate. When comparing the co-receptor subunits (Fig. 3B), we observed that the S1-domain arginine is conserved in CpomIR25a (Arg512) but replaced by a glutamine in CpomIR76b (Gln135). In contrast, the S2-domain negatively-charged residue is conserved in both co-receptors: as an aspartate (Asp762) in CpomIR25a and as a glutamate (Glu336) in CpomIR76b, mirroring the Glu480 residue in CpomIR41a1 (Glu480) (Fig. 3C). The presence of these conserved glutamate-binding residues in both CpomIR41a1 and the co-receptors is compelling. It raises the hypothesis that the conserved negatively charged residues in the S2 domain may contribute to non-covalent interaction with the amino groups of the polyamines we identified as ligands (Figs. 1, 2). This idea is further supported by the apparent absence of these residues in subunits that bind acids (Supplementary Figure S4). Future studies involving site-directed mutagenesis, targeting the IR coding sequences using methods similar to those previously applied to CpomOrco [57], may reveal whether these residues are indeed responsible for ligand binding specificity.

Another broadly conserved IR is IR75d, which belongs to the IR75 clade and maintains a clear one-to-one orthologous relationship across dipteran and lepidopteran lineages [3, 17, 27, 51]. In the *D. melanogaster* genome, the IR75 clade is composed of DmelIR75d and three other paralogs, DmelIR75a, DmelIR75b and DmelIR75c. DmelIR75d is activated by pyrrolidine whereas the other IR75s by C2-C6 carboxylic acids [10, 16, 17, 20–22]. Similarly, other insect lineages have their own specific IR75 paralogs beside IR75d [3, 27, 51]. Several of these IR75 subunits have been functionally characterized, showing tuning to carboxylic acids. For instance, AaegIR75k1 and AaegIR75k3 from *Aedes aegypti*, as well as AalbIR75e from *Aedes albopictus*, respond to C7-C9 carboxylic acids [24], and the agonists of *A. gambiae* AgIR75k are C6-C10 carboxylic acids [25]. Lepidopteran IRs AsegIR75p.1 and AsegIR75q.1 from *Agrotis segetum* also respond to medium-chain fatty acids, with hexanoic acid being the most potent agonist for AsegIR75p.1 [27]. In our screening (Fig. 1D), we were unable to evaluate the conservation of pyrrolidine-evoked responses as described for DmelIR75d, as the firing activity conferred by DmelIR76a-expressing OSNs to ac4 sensilla masked the response of transgenic DsuzIR75d [10, 11, 17]. However, we clearly identified hexanoic acid as an agonist for DsuzIR75d. This suggests a conserved functional specialization for carboxylic acids that originated in the ancestral IR75 receptor and that has been lost in DmelIR75d. In support of this hypothesis, both DsuzIR75d and DmelIR75d display the same aforementioned arginine (Fig. 3) proximal to the end of the LBD-S1 domain (Fig. 4), which is conserved in iGluRs [4, 8] and acid-sensing IRs, and it is thought to be important for carboxyl group binding [12, 42]. Interestingly, structural analysis unveiled this arginine protruding towards the S1/S2 binding pocket of the LBD (Fig. 4C). The fact that DmelIR75d does not respond to acids, but it maintains this amino acid feature key for acid sensing may be a clue of its origin from an ancestral acid sensor.

When analyzing the LBD of IR75d in both *D. melanogaster* and *D. suzukii* (Fig. 4), we identified five conserved amino acid substitutions and a non-conserved substitution in the S2 domain (Gln/Arg478). Structural analysis revealed that despite being part of the S2 sequence, the non-conserved residue projects externally to the LBD, in contrast to the residues corresponding to the conserved amino acid substitutions, which are located closer to the S1-S2 binding pocket. The presence of a positively charged residue in this position is shared among all acid-sensing IR-subunits, except for DmelIR31a (Supplementary Figure S4), and it may play a role in the binding of carboxylic acids by DsuzIR75d. Further investigation is warranted to uncover the potential roles of these amino acids in the functional switch of DmelIR75d sensitivity from acids to polyamines. With the advent of CRISPR-gene editing in *Drosophila* [58], this hypothesis can be tested by replacing IR75d-variants directly *in vivo*. Additionally, a broader investigation aimed at testing the sensitivity of IR75d orthologs across different dipterans would shed light on whether the switch to polyamines is specific to *D. melanogaster* or if both responses can coexist for the same IR subunits in other insects.

While being able to deorphanize CpomIR41a by expressing it along a native *D. melanogaster* IR, and DsuzIR75d by expressing it in a novel “ionotropic receptor decoder” in an empty DmelIR25a-based OSN, we failed to identify ligands for DsuzIR64a expressed in the “ionotropic receptor decoder” system used in previous studies [10, 14, 20–22] (Fig. 1F). In fact, transgenic ac4 sensilla had no new firing activity compared to the control knock-out line. This suggests that either the IR64a-transgene expression was not functional, or the specific activating ligands were not present in the odor panel used for screening. In *D. melanogaster*, DmelIR64a is activated by hydrochloric acid and acetic acid [13, 17, 19, 59] therefore if transgenic DsuzIR64a was indeed functionally expressed, a shift in its specificity has occurred in *D. suzukii* relative to *D. melanogaster*. When we examined these genes at the sequence level (Fig. 5), we observed that the LBD from both *Drosophila* species was mostly identical except for six substitutions, three out of which (Ser416Leu in the S1 and Ser691Pro and Ser712 Ala) were not conserved. However, a structural analysis demonstrated these substitutions are positioned outside the S1/S2 pocket of the LBD potentially excluding their possible involvement in ligand binding (Supplementary Figure S5). In contrast, the Arg402 present in all acid-sensing IRs and iGluRs [12, 42] protruded towards the binding pocket. We additionally observed a deletion of three consecutive glutamines within the intracellular loop 1 of the ion pore channel of DsuzIR64a subunit. It is known that in other olfactory cation channels in insects, small changes in the amino acid sequence of intracellular loops may influence the ligand binding ability and its pharmacology [57, 60–62]. Future comparative studies may address the various differences that we have observed in the sequence of DsuzIR64a, with the aim to understand their possible influences in ligand binding.

Upon expression of DsuzIR64a or a truncated CpomIR64a isoform in the “ionotropic receptor decoder” neuron (Fig. 1G), the firing frequency of ac4 sensilla decreased when stimulated with pyrrolidine, dimethylamine, and ammonium hydroxide compared to the control knockout line. This reduction in stimulation may be associated with the expression of heterologous subunits, either functional or not. We are aware that this effect was not observed during previous studies that used the “ionotropic receptor decoder” neuron [8, 21, 22]. However, these studies did not test a wide panel of ligands such as in our study but rather focused on effects of specific compounds. A similar phenomenon was also observed in the transgenic line expressing CpomIR41a1, which inhibited the responses of ac4-neurons to phenylacetaldehyde, dimethylamine and phenylacetic acid. These ligands activate DmelIR84-expressing OSNs [14, 17], which were not the target of CpomIR41a1 transgene expression. Similarly, the expression of DsuzIR75d inhibited ac4 response to butylamine, which was detectable in the corresponding knock-out control line. If on one side, the expression of a heterologous IR may reduce tuning to ligands for the IRs expressed in other neurons, as in “opponent signaling” [17] where agonists for one OSN antagonized the activity in another, our observations add a new dimension to this phenomenon, since we observed the same effect when we also expressed the truncated CpomIR64a∆. Considering this fact, future studies are definitely needed to more thoroughly understand why and how some ectopically or heterologously expressed IR tuning subunits alter the firing responses of other OSNs housed in the same sensillum.

Finally, we also explored heterologous expressions in HEK cells (Fig. 6), which is a single-cell ex vivo system, such as *Xenopus* oocytes. HEK cells have been largely used in the deorphanization of ORs [46, 57, 63–66] whereas, to our knowledge, this method has not been previously utilized for IR subunit deorphanization. We conducted several experiments using this approach but did not achieve successful deorphanization of IRs, despite our immunohistochemical results suggesting a correct expression and targeting of both DsuzIR8a and a DmelIR8a control, when expressed in HEK cells (Fig. 6A). This may be attributed to several factors. Whether the lack of IR-functionality is potentially determined by their overall expression level or rather their possible incorrect assembly we do not know. Although possibly, the distinct cellular environment of HEK cells compared to OSNs could contribute to the differences in receptor function and signaling. Additionally, the poor surface localization of IR proteins in HEK cells could be a limiting factor, as it has been observed in HEK cells expressing ORs where proteins were retained in intracellular membranes hindering their proper functioning and interaction with ligands [64, 67]. These results highlight the challenges associated with the heterologous expression approach in HEK cells for IR deorphanization. Further studies are needed to optimize this method and address the limitations encountered.

## Conclusions

Adding to previous efforts conducted to functionally characterize insect IRs by heterologous expression, our study illustrates that the replacement of tuning IRs in OSNs of *D. melanogaster* with IRs from distant lineages, as well as the expression of these alongside the native *Drosophila* IRs, leads to the formation of functional heteromeric complexes. For the first time, we have used *D. melanogaster* to deorphanize IR subunits of insects not belonging to its close phylogeny. This achievement not only contributes to the ongoing deorphanization endeavors concerning chemoreceptors in pests like *C. pomonella* and *D. suzukii* but also holds the potential to guide future investigations into IRs from various other species. Once more, the successful use of *D. melanogaster* has proved instrumental in advancing methodologies aimed at the study of insect chemoreceptors. Our results incorporate the use of transgenic expression in *D. melanogaster* into the toolkit of methods for in vivo functional analysis of heterologous IR subunits, spanning from ligand binding to pharmacology.

## Supplementary Information

Supplementary file 7. Figure S4. Supplementary polypeptide sequence alignment of IR75d orthologues together with acid sensing subunits. In the dataset we included the following: DsuzIR75a; DsuzIR75b [33]; DsuzIR75c; DsuzIR75d and DsuzIR64a; DmelIR75b; DmelIR75c; DmelIR75d; DmelIR64a; DmelIR84a; DmelIR31a Isoform C; DmelIR8a; DsecIR75a; DsecIR75b; DsecIR75c; DsecIR75d; DsecIR64a; CpomIR64a. Sequences were aligned using Muscle software. Alignment accuracy was checked manually and refined using BioEdit v7.2.5 [44]. Lilac filled square: S1 domain; light-blue filled square: S2 domains, according to Prieto-Godino et al. [21]. Green square: S2-CREL according with Abuin et al. [9]. Note: Prieto-Godino et al. [21] used a variant of DmelIR75b, which is 4 amino acids longer and no PDB-files are available for this variant. Black squares: transmembrane domains based on TopCons. Orange squares: amino acid substitutions identified within the LBD of DmelIR75d, when compared with the *D. suzukii* orthologue. Brown squares: amino acid substitutions, insertions and mutations between the IR64a orthologues of *D. melanogaster* and* D. suzukii*. Red square: arginine proximal to the end of the LBD-S1 domain, which is conserved in iGluRs and acid-sensing IRs, assigned for carboxyl group binding [12, 42]. With the red square we have also highlighted an additional positively-charged residue we identified at the beginning of the LBD-S2 domain, which is conserved in all acid sensing IRs, except for IR31a.

Supplementary file 8. Figure S5 Protein model based on DmelIR64a generated by RasTop and magnified view of the ligand-binding domain. Lilac ribbons: S1-LBD; light-blue ribbons: S2-LBD. Red residues: positions of the conserved Arg402 [12, 42], and of the Lys623 residuethat in Supplementary Figure S4 we have indicated to be conserved among most of the acid sensing IRs. Yellow residues: amino acid substitutions within the S1/S2 between the *D. melanogaster* and *D. suzukii* orthologues, as indicated in Figure 5: Val/Leu312 and Leu/Ser414 in the S1, Ser/Thr632, Pro/Ser689, Ala/Ser710 and Thr/Ser791 in the S2. Note: the PDB accession Q9 VRL4 provides a protein, which amino acid at position 414 is a Serine as in its *D. suzukii* orthologue. Note: except for Arg402, all of the indicated residues are distant from the S1/S2 pocket of the LBD.

Supplementary file 9. Figure S6 Summary diagram of the *pros* and *cons* from the use of heterologous methods based either on *Drosophila* transgenic neurons or HEK293 cells, when attempting the functional characterization of insect chemosensory cation channels like IRs.

Supplementary file 10. Table S1. Primers used to amplify ORFs. The *attB* regions added upstream are reported below. Kozak CACC sequences on forward primers [46] are indicated in bold. Melting temperatures were calculated by Oligocalc, submitting sequences without *attB*-regions, and selecting the salt adjusted optimization.

Supplementary file 11. Table S2 SPSS outputs for spike counting and neuronal counting. When conducting tests of normality, datasets not normally distributed are highlighted in yellow. Evidence that in every fly-line tested, datasets were not normally distributed for at least one case-ligand resulted in conducting a non-parametric statistical analysis despite for the fly line *w;UASCpomIR64a∆;IR84a-Gal4*^*KI*^ all datasets were normally distributed. For comparative reasons, Supplementary Data File 1 provides also a parametric statistical analysis.

Supplementary file 12. Table S3 List of PDB-accession from the polypeptide sequence aligned with CpomIR41a1. The table shows calculation of sequence identity and similarity. Presence of the S1/S2-key residues for iGluRs’ binding of the glutamate ligand are indicated.

Supplementary file 1. Raw spike counting and bar charts of ac4-enhanced activation from various transgenic flies. Control fly line genotypes: Oregon-C wild type, *w;IR75d-Gal4;IR75d*^*KO*^, *w;UAS-IR75d*^*HEK*^*;IR75d*^*KO*^and *w;Bl/CyO;IR84a-Gal4*^*KI*^. Fly-line genotypes expressing heterologous IRs: *w;IR76a-Gal4;UAS-CpomIR41a1*, *w;IR75d-Gal4/UAS-DsuzIR75d*^*HEK*^^*K*^*;IR75d*^*KO*^, *w;UAS-DsuzIR64a;IR84a-Gal4*^*KI*^, and *w;UAS-CpomIR64a∆;IR84a-Gal4*^*KI*^. Choice of p-values from a parametric or a non-parametric statistical analysis depended on tests for normal distribution. The magnitude of p-values has been distinguished as it follows: * *p* < 0.05; ** 0.001 < *p* < 0.01; *** *p* < 0.001; which is consistent with Figure 1. Note: colors have been used to distinguish genotypes and remark their significant effects based on colors used in Figure 1. For the tested ligands, blue names indicate ligands diluted in water and red ligands diluted in ethanol.

Supplementary file 2. Dose-response experiments for CpomIR41a1 and DsuzIR75d.Stimulus were prepared by diluting compounds in water at different % vol/vol [0.1-3.0] and using 1.0-30 μL aliquots of such dilutions on circle filter paper, preparing a serial set of doses [0.1-90]. Based on respective volumes of neat compound content per stimulus, moles of putrescine, spermidine and hexylamine per doses were calculated based on their densities and molecular weights. Raw data reports ∆spikes/0.5 sec frequencies per dose at each replicate. Data were normalized to the effect of dose 20 for putrescine.Stimuli were prepared by diluting compounds in ethanol at different % vol/vol [0.1-3.0] and using 1.0-20 μL aliquots of such dilutions on circle filter paper, preparing a serial set of doses [0.15-90]. Based on respective volumes of neat compound content per stimulus, moles of hexanoic acid, trimethylamine and butylamine per doses were calculated based on their densities and molecular weights. Raw data reports ∆spikes/0.5 sec frequencies per dose at each replicate. Note: we were not able to establish a precise saturating dose, which for some replicates resulted at DOSE 10 and in others at DOSE 20.

Supplementary file 3. Neuronal counting on *D. suzukii* male and female antennae stained with IR64a-probes. Choice of a parametric statistical analysis depended on tests for normal distribution. The file shows neuronal counting statistical parameters from a Heteroscedastic Two-sample T-testand parameters from the box plot analysis of the number of counted neurons.

Supplementary file 4. Figure S1. Schematic representation of crossings to generate transgenic lines for SSR. Note: for the various transgenic lines, the transgene is indicated in bold

Supplementary file 5. Figure S2 Supplementary polypeptide sequence alignment of IR41a1-orthologues and DmelIR25a, DmelIR76b co-receptors. S1/S2 domains, S1-arginine and S2-negatively charged residue are indicated as in Figure 3. Amino acid positions have been BioEdit-adjusted based on findings from Benton et al. [8]. Green square depicts the IR25a S2-CREL as in Abuin et al. [9].

Supplementary file 6. Figure S3. Dose-response effects testing trimethylamine and butylamine on DsuzIR75d.Spike trains of ac4 generated by DOSE 20 of trimethylamine and butylamine. Red bar: stimulus. Dose-response characteristics of ac4 sensilla recorded from antennae of *w;IR75d-Gal4/UAS-DsuzIR75d*^*HEK*^*;IR75d*^*KO*^ fly lines to trimethylamineand butylamineexpressed as a function of spike frequency. Right: summary plot.

## Data Availability

The original contributions presented in the study are included in the article and in the supplementary material. A previous version of this manuscript has been deposited on BioRXiv with the following DOI: 10.1101/2023.09.12.557369. Further inquiries can be directed to the corresponding author.

## Abbreviations

IR: Ionotropic receptors
Cpom: *Cydia pomonella*
Dsuz: *Drosophila suzukii*
Dmel: *Drosophila melanogaster*
Dsec: *Drosophila sechellia*
SSR: Single sensillum recording
Fish: Fluorescent in situ hybridization
LBD: Ligand binding domain
PDB: Protein data bank
Orco: Olfactory receptor co-coreceptor
HEK: Human embryonic kidney cells
CpomIR64a∆: Truncated ORF of CpomIR64a
