# Supplementary figures and images for: Alternative strategies based on transgenic Drosophila melanogaster for the functional characterization of insect Ionotropic Receptors
Source: Biol Res. 2025 Jun 9;58:36. doi: 10.1186/s40659-025-00619-0 (PMC12147327; doi:10.1186/s40659-025-00619-0)

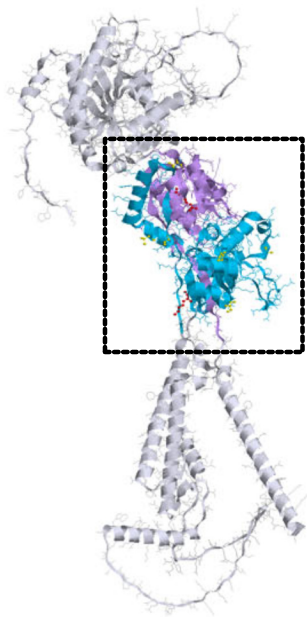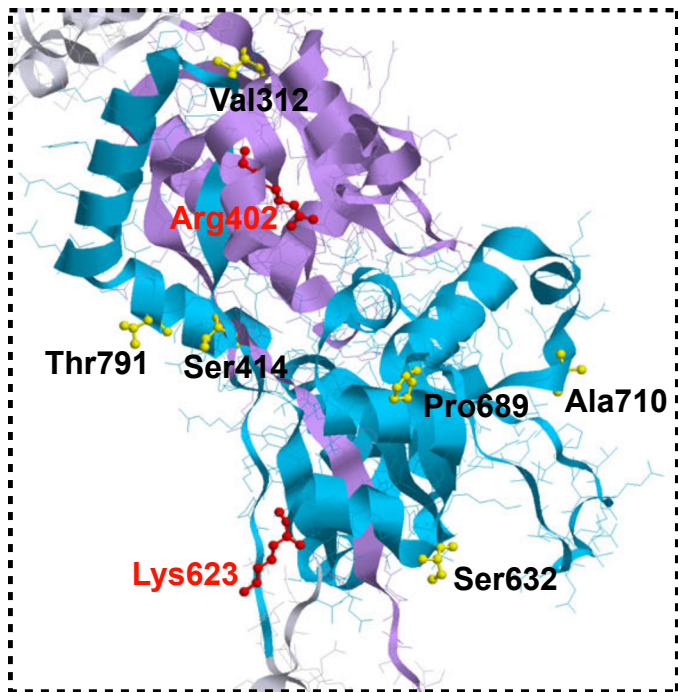

Supplement: Supplementary file 2 — Supplementary file 8. Figure S5 Protein model based on DmelIR64a generated by RasTop and magnified view of the ligand-binding domain. Lilac ribbons: S1-LBD; light-blue ribbons: S2-LBD. Red residues: positions of the conserved Arg402 [12, 42], and of the Lys623 residuethat in Supplementary Figure S4 we have indicated to be conserved among most of the acid sensing IRs. Yellow residues: amino acid substitutions within the S1/S2 between the D. melanogaster and D. suzukii orthologues, as indicated in Figure 5: Val/Leu312 and Leu/Ser414 in the S1, Ser/Thr632, Pro/Ser689, Ala/Ser710 and Thr/Ser791 in the S2. Note: the PDB accession Q9 VRL4 provides a protein, which amino acid at position 414 is a Serine as in its D. suzukii orthologue. Note: except for Arg402, all of the indicated residues are distant from the S1/S2 pocket of the LBD. [file 40659_2025_619_MOESM2_ESM.pdf]

A

Triethylamine

Butylamine

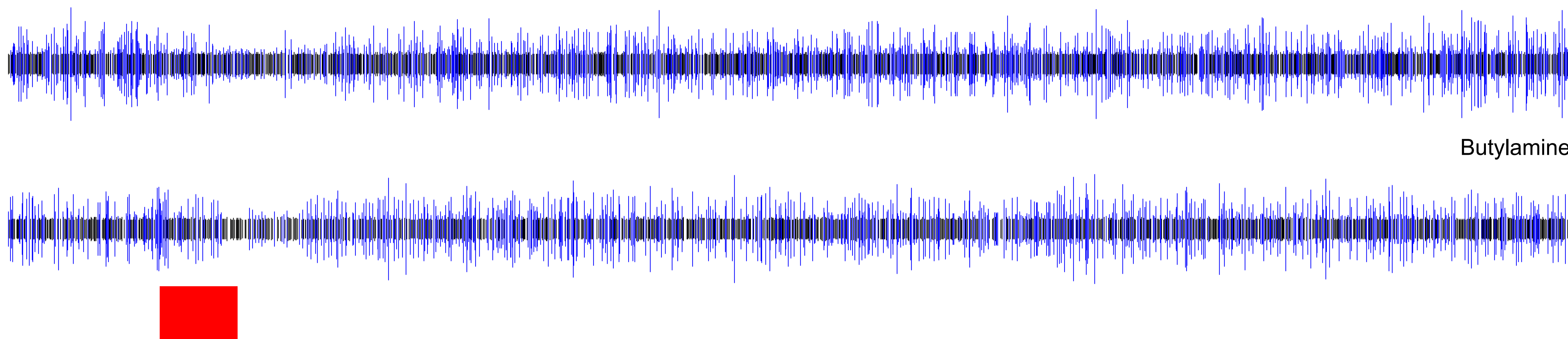

B

Triethylamine

Butylamine

Summary plot

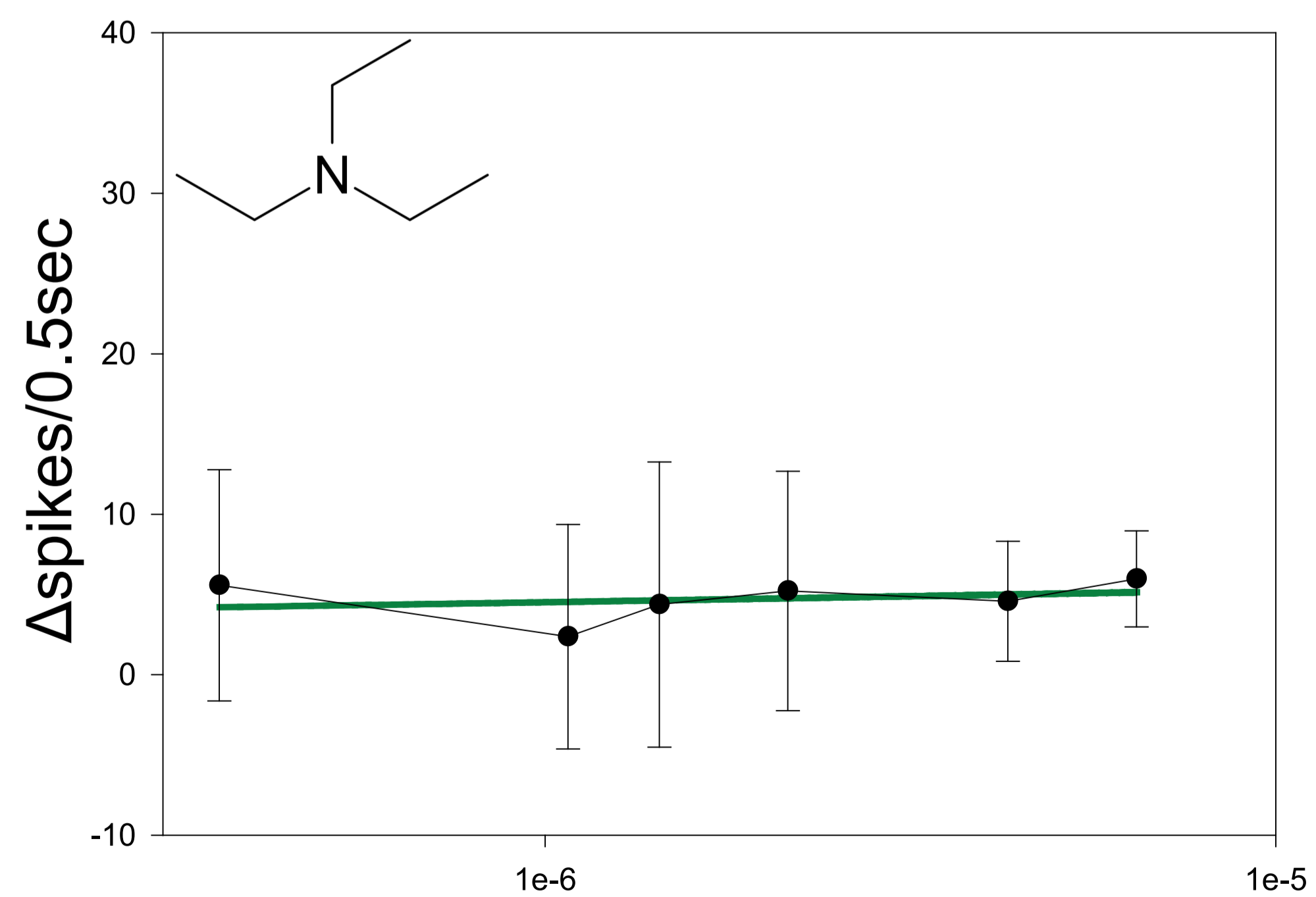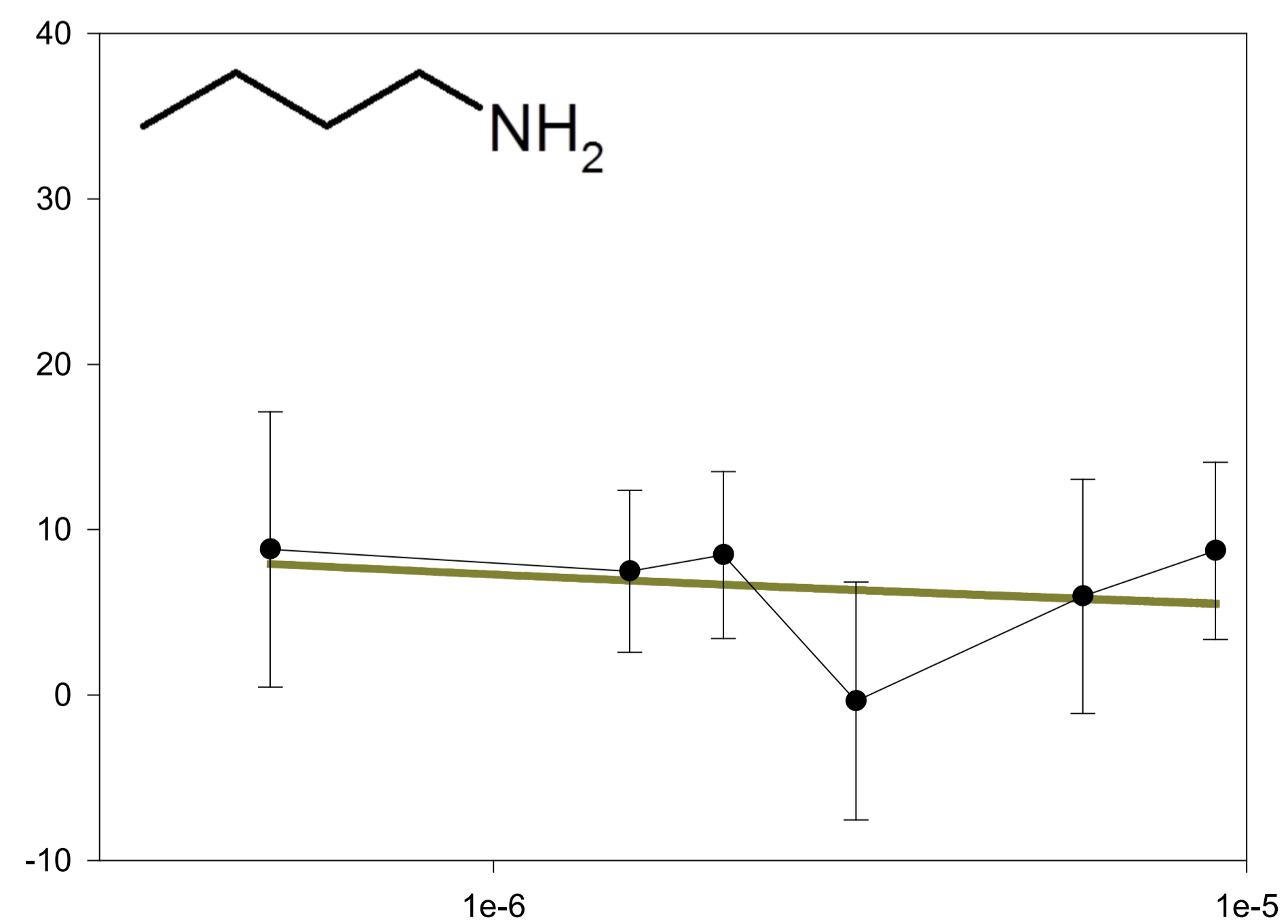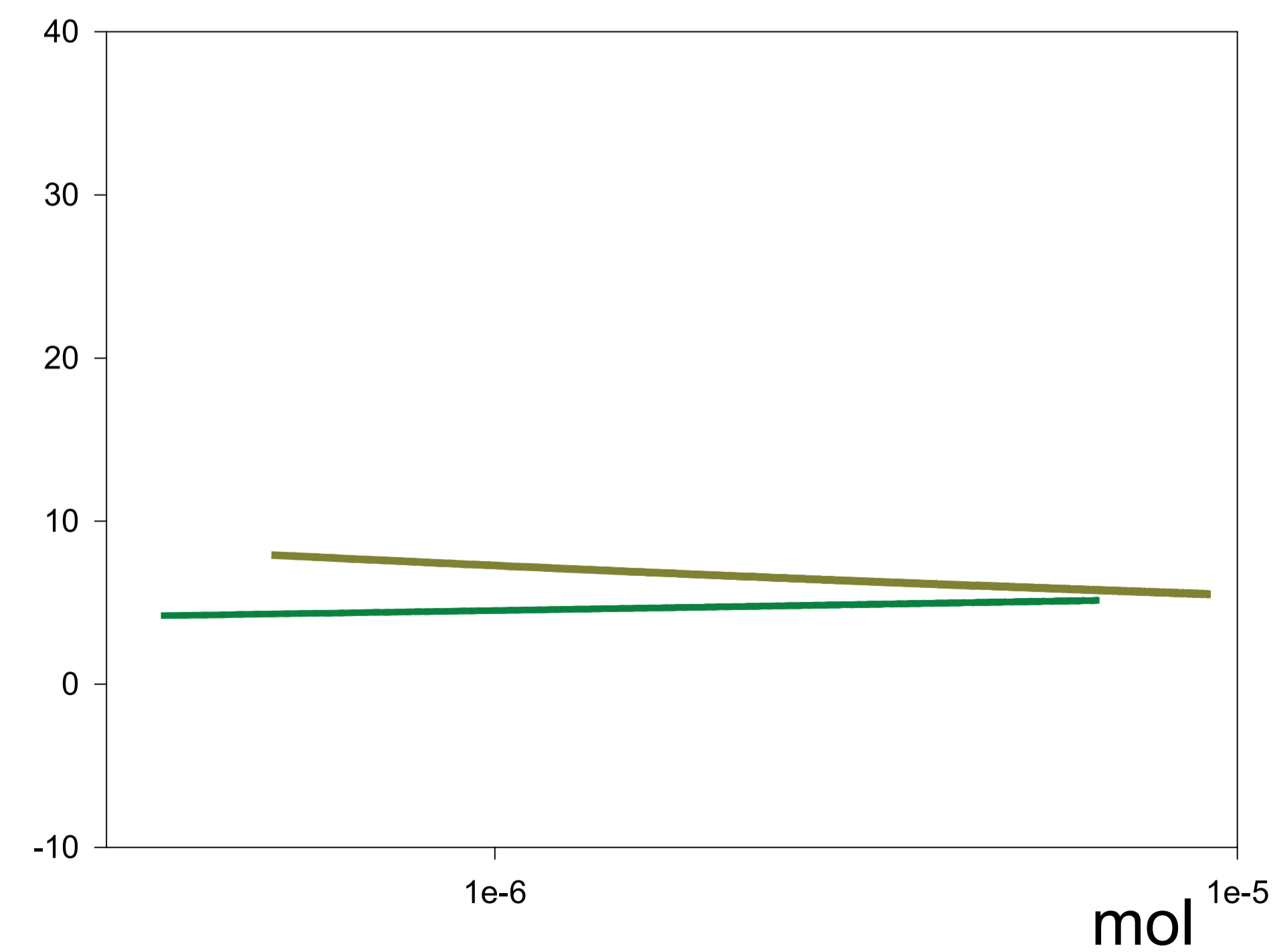

Supplement: Supplementary file 12 — Supplementary file 6. Figure S3. Dose-response effects testing trimethylamine and butylamine on DsuzIR75d.Spike trains of ac4 generated by DOSE 20 of trimethylamine and butylamine. Red bar: stimulus. Dose-response characteristics of ac4 sensilla recorded from antennae of w;IR75d-Gal4/UAS-DsuzIR75dHEK;IR75dKO fly lines to trimethylamineand butylamineexpressed as a function of spike frequency. Right: summary plot. [file 40659_2025_619_MOESM12_ESM.pdf]
