# Supplementary material for: Alternative strategies based on transgenic Drosophila melanogaster for the functional characterization of insect Ionotropic Receptors
Source: Biol Res. 2025 Jun 9;58:36. doi: 10.1186/s40659-025-00619-0 (PMC12147327; doi:10.1186/s40659-025-00619-0)
Supplement: Supplementary file 3 — Supplementary file 9. Figure S6 Summary diagram of the pros and cons from the use of heterologous methods based either on Drosophila transgenic neurons or HEK293 cells, when attempting the functional characterization of insect chemosensory cation channels like IRs. [file 40659_2025_619_MOESM3_ESM.pdf]

D. melanogaster neurons

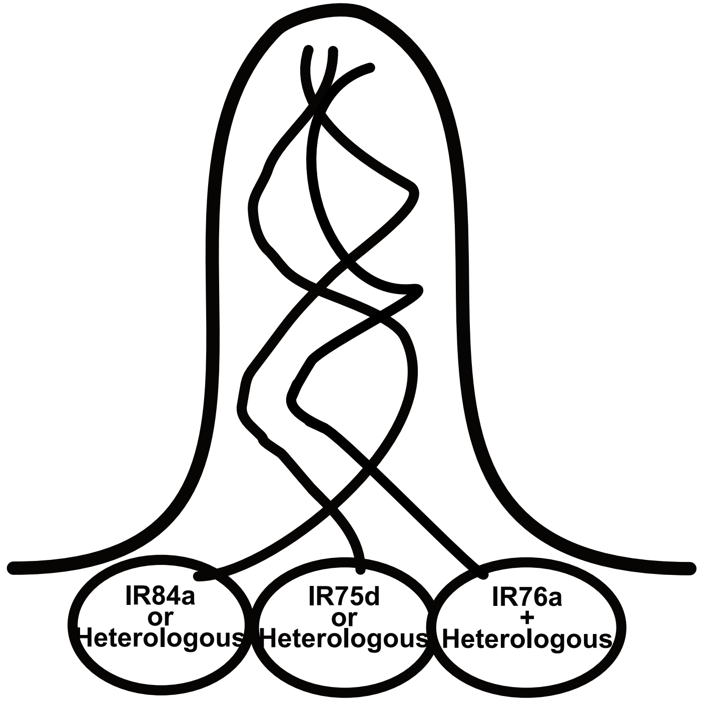

HEK293 cells

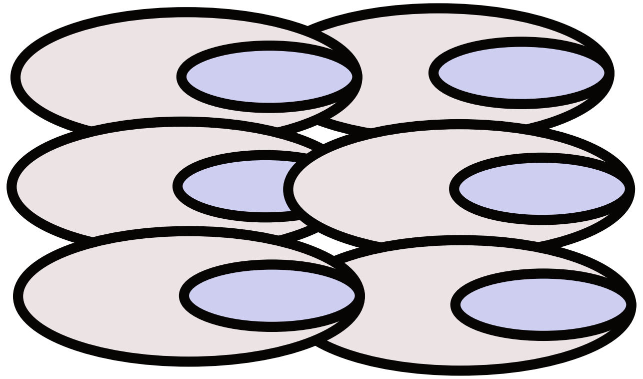

	Pros	Cons
D. melanogaster neurons	- Similar intracellular environement (co-receptors/co-factors)<br>- Similar extracellular environement (olfactory binding proteins)<br>- More realistic physicochemical environment for odor delivery (vapor)<br>- Increased sensitivity/high fidelity receptive field for the heterologous chemoreceptor	- Time consuming (multiple crossing)<br>- Insects need to be reared<br>- Not suitable for high throughput screening (HTS)<br>- Interferences with the ligand sensitivity from local neurons (biased effects)
HEK293 cells	- Fast and easy to transfect (transient or stable)<br>- If stable, transgenic cells can be stocked for long term<br>- Scalable to high throughput screening<br>- Accessable to electrophysiology and fluorimetry upon needs: combination of both methods	- Diverse intracellular environment (issue with receptor folding and targeting)<br>- Needs of complex co-expression methods to combine various subunits of the same receptor<br>- Less realistic physicochemical environement for odor delivery (liquid)
