# Supplementary material for: Alternative strategies based on transgenic Drosophila melanogaster for the functional characterization of insect Ionotropic Receptors
Source: Biol Res. 2025 Jun 9;58:36. doi: 10.1186/s40659-025-00619-0 (PMC12147327; doi:10.1186/s40659-025-00619-0)
Supplement: Supplementary file 5 — Supplementary file 11. Table S2 SPSS outputs for spike counting and neuronal counting. When conducting tests of normality, datasets not normally distributed are highlighted in yellow. Evidence that in every fly-line tested, datasets were not normally distributed for at least one case-ligand resulted in conducting a non-parametric statistical analysis despite for the fly line w;UASCpomIR64a∆;IR84a-Gal4KI all datasets were normally distributed. For comparative reasons, Supplementary Data File 1 provides also a parametric statistical analysis. [file 40659_2025_619_MOESM5_ESM.docx]

Spike counting

| **Tests of Normality -** *Oregon, WT* | | | | | | |
| --- | --- | --- | --- | --- | --- | --- |
|  | Kolmogorov-Smirnov^a^ | | | Shapiro-Wilk | | |
|  | Statistic | df | Sig. | Statistic | df | Sig. |
| Phenylacetaldehyde (ac4) | ,133 | 6 | ,200^*^ | ,986 | 6 | ,978 |
| 3-octanol (ac3) | ,269 | 6 | ,200^*^ | ,879 | 6 | ,266 |
| Pyridine (ac2) | ,188 | 6 | ,200^*^ | ,924 | 6 | ,532 |
| Putrescine (ac2) | ,204 | 6 | ,200^*^ | ,906 | 6 | ,408 |
| Pyrrolidine (ac1/ac2/ac4) | ,233 | 6 | ,200^*^ | ,868 | 6 | ,220 |
| Dimethylamine (ac1/ac4) | ,223 | 6 | ,200^*^ | ,921 | 6 | ,509 |
| Ammonium hydroxide (ac1/ac4) | ,260 | 6 | ,200^*^ | ,854 | 6 | ,171 |
| Phenylaceic acid (ac4) | ,190 | 6 | ,200^*^ | ,938 | 6 | ,640 |
| 2-phenyletylamine (ac1/ac4) | ,166 | 6 | ,200^*^ | ,928 | 6 | ,564 |
| Ammonia (ac1/ac4) | ,187 | 6 | ,200^*^ | ,969 | 6 | ,884 |
| Cadaverine (ac2) | ,192 | 6 | ,200^*^ | ,972 | 6 | ,908 |
| Spermidine (ac2) | ,256 | 6 | ,200^*^ | ,849 | 6 | ,154 |
| Hexylamine | ,185 | 6 | ,200^*^ | ,975 | 6 | ,927 |
| Triethylamine | ,353 | 6 | ,019 | ,778 | 6 | ,037 |
| Buthylamine | ,249 | 6 | ,200^*^ | ,882 | 6 | ,279 |
| Benzaldehyde | ,144 | 6 | ,200^*^ | ,981 | 6 | ,958 |
| Beta citronellol | ,223 | 6 | ,200^*^ | ,965 | 6 | ,861 |
| Formic acid | ,324 | 6 | ,048 | ,797 | 6 | ,055 |
| Acetic acid (ac2) | ,413 | 6 | ,002 | ,636 | 6 | ,001 |
| Propionic acid | ,345 | 6 | ,025 | ,772 | 6 | ,032 |
| Butanoic acid | ,340 | 6 | ,028 | ,779 | 6 | ,038 |
| Hexanoic acid | ,312 | 6 | ,069 | ,793 | 6 | ,051 |
| Octanoi acid | ,199 | 6 | ,200^*^ | ,904 | 6 | ,398 |
| Solvent - Water | ,258 | 6 | ,200^*^ | ,946 | 6 | ,711 |
| Solvent - Ethanol | ,192 | 6 | ,200^*^ | ,971 | 6 | ,899 |
| *. This is a lower bound of the true significance. | | | | | | |
| a. Lilliefors Significance Correction | | | | | | |
| **Tests of Normality -** *w;pIR76a-Gal4;pUASCpomIR41a1* | | | | | | |
|  | Kolmogorov-Smirnov^a^ | | | Shapiro-Wilk | | |
|  | Statistic | df | Sig. | Statistic | df | Sig. |
| Phenylacetaldehyde (ac4) | ,283 | 6 | ,145 | ,830 | 6 | ,108 |
| 3-octanol (ac3) | ,299 | 6 | ,101 | ,863 | 6 | ,201 |
| Pyridine (ac2) | ,281 | 6 | ,150 | ,874 | 6 | ,243 |
| Putrescine (ac2) | ,279 | 6 | ,158 | ,888 | 6 | ,308 |
| Pyrrolidine (ac1/ac2/ac4) | ,256 | 6 | ,200^*^ | ,925 | 6 | ,543 |
| Dimethylamine (ac1/ac4) | ,194 | 6 | ,200^*^ | ,940 | 6 | ,656 |
| Ammonium hydroxide (ac1/ac4) | ,133 | 6 | ,200^*^ | ,982 | 6 | ,959 |
| Phenylaceic acid (ac4) | ,229 | 6 | ,200^*^ | ,846 | 6 | ,146 |
| 2-phenyletylamine (ac1/ac4) | ,287 | 6 | ,134 | ,818 | 6 | ,084 |
| Ammonia (ac1/ac4) | ,270 | 6 | ,195 | ,891 | 6 | ,321 |
| Cadaverine (ac2) | ,416 | 6 | ,002 | ,651 | 6 | ,002 |
| Spermidine (ac2) | ,254 | 6 | ,200^*^ | ,902 | 6 | ,386 |
| Hexylamine | ,273 | 6 | ,183 | ,882 | 6 | ,279 |
| Triethylamine | ,253 | 6 | ,200^*^ | ,876 | 6 | ,249 |
| Buthylamine | ,174 | 6 | ,200^*^ | ,933 | 6 | ,602 |
| Benzaldehyde | ,212 | 6 | ,200^*^ | ,933 | 6 | ,607 |
| Beta citronellol | ,193 | 6 | ,200^*^ | ,963 | 6 | ,844 |
| Formic acid | ,349 | 6 | ,021 | ,758 | 6 | ,024 |
| Acetic acid (ac2) | ,201 | 6 | ,200^*^ | ,975 | 6 | ,924 |
| Propionic acid | ,382 | 6 | ,007 | ,766 | 6 | ,029 |
| Butanoic acid | ,252 | 6 | ,200^*^ | ,866 | 6 | ,209 |
| Hexanoic acid | ,332 | 6 | ,037 | ,770 | 6 | ,031 |
| Octanoi acid | ,151 | 6 | ,200^*^ | ,961 | 6 | ,830 |
| Solvent - Water | ,295 | 6 | ,112 | ,866 | 6 | ,210 |
| Solvent - Ethanol | ,243 | 6 | ,200^*^ | ,948 | 6 | ,724 |
| *. This is a lower bound of the true significance. | | | | | | |
| a. Lilliefors Significance Correction | | | | | | |

| **Tests of Normality** **-** *w;pIR75d-Gal4;IR75^KO^* | | | | | | |
| --- | --- | --- | --- | --- | --- | --- |
|  | Kolmogorov-Smirnov^a^ | | | Shapiro-Wilk | | |
|  | Statistic | df | Sig. | Statistic | df | Sig. |
| Phenylacetaldehyde (ac4) | ,175 | 7 | ,200^*^ | ,957 | 7 | ,794 |
| 3-octanol (ac3) | ,214 | 7 | ,200^*^ | ,862 | 7 | ,158 |
| Pyridine (ac2) | ,257 | 7 | ,180 | ,891 | 7 | ,279 |
| Putrescine (ac2) | ,268 | 7 | ,138 | ,883 | 7 | ,239 |
| Pyrrolidine (ac1/ac2/ac4) | ,164 | 7 | ,200^*^ | ,942 | 7 | ,653 |
| Dimethylamine (ac1/ac4) | ,259 | 7 | ,170 | ,809 | 7 | ,050 |
| Ammonium hydroxide (ac1/ac4) | ,212 | 7 | ,200^*^ | ,909 | 7 | ,392 |
| Phenylaceic acid (ac4) | ,230 | 7 | ,200^*^ | ,884 | 7 | ,244 |
| 2-phenyletylamine (ac1/ac4) | ,208 | 7 | ,200^*^ | ,907 | 7 | ,379 |
| Ammonia (ac1/ac4) | ,235 | 7 | ,200^*^ | ,909 | 7 | ,391 |
| Cadaverine (ac2) | ,180 | 7 | ,200^*^ | ,922 | 7 | ,482 |
| Spermidine (ac2) | ,178 | 7 | ,200^*^ | ,961 | 7 | ,823 |
| Hexylamine | ,338 | 7 | ,015 | ,823 | 7 | ,068 |
| Triethylamine | ,332 | 7 | ,019 | ,833 | 7 | ,086 |
| Buthylamine | ,201 | 7 | ,200^*^ | ,949 | 7 | ,716 |
| Benzaldehyde | ,198 | 7 | ,200^*^ | ,934 | 7 | ,582 |
| Beta citronellol | ,203 | 7 | ,200^*^ | ,930 | 7 | ,554 |
| Formic acid | ,162 | 7 | ,200^*^ | ,976 | 7 | ,940 |
| Acetic acid (ac2) | ,216 | 7 | ,200^*^ | ,920 | 7 | ,467 |
| Propionic acid | ,203 | 7 | ,200^*^ | ,905 | 7 | ,359 |
| Butanoic acid | ,340 | 7 | ,014 | ,800 | 7 | ,041 |
| Hexanoic acid | ,227 | 7 | ,200^*^ | ,919 | 7 | ,461 |
| Octanoi acid | ,176 | 7 | ,200^*^ | ,943 | 7 | ,666 |
| Solvent - Water | ,155 | 7 | ,200^*^ | ,960 | 7 | ,822 |
| Solvent - Ethanol | ,184 | 7 | ,200^*^ | ,963 | 7 | ,841 |
| *. This is a lower bound of the true significance. | | | | | | |
| a. Lilliefors Significance Correction | | | | | | |

| **Tests of Normality -** *w;pUASDsuzIR75d^HEK^;IR75^KO^* | | | | | | |
| --- | --- | --- | --- | --- | --- | --- |
|  | Kolmogorov-Smirnov^a^ | | | Shapiro-Wilk | | |
|  | Statistic | df | Sig. | Statistic | df | Sig. |
| Phenylacetaldehyde (ac4) | ,161 | 6 | ,200^*^ | ,979 | 6 | ,945 |
| 3-octanol (ac3) | ,209 | 6 | ,200^*^ | ,883 | 6 | ,282 |
| Pyridine (ac2) | ,198 | 6 | ,200^*^ | ,956 | 6 | ,787 |
| Putrescine (ac2) | ,290 | 6 | ,124 | ,866 | 6 | ,213 |
| Pyrrolidine (ac1/ac2/ac4) | ,201 | 6 | ,200^*^ | ,915 | 6 | ,468 |
| Dimethylamine (ac1/ac4) | ,199 | 6 | ,200^*^ | ,873 | 6 | ,239 |
| Ammonium hydroxide (ac1/ac4) | ,204 | 6 | ,200^*^ | ,914 | 6 | ,464 |
| Phenylaceic acid (ac4) | ,300 | 6 | ,098 | ,874 | 6 | ,243 |
| 2-phenyletylamine (ac1/ac4) | ,236 | 6 | ,200^*^ | ,903 | 6 | ,390 |
| Ammonia (ac1/ac4) | ,343 | 6 | ,026 | ,711 | 6 | ,008 |
| Cadaverine (ac2) | ,191 | 6 | ,200^*^ | ,915 | 6 | ,472 |
| Spermidine (ac2) | ,247 | 6 | ,200^*^ | ,933 | 6 | ,600 |
| Hexylamine | ,211 | 6 | ,200^*^ | ,907 | 6 | ,414 |
| Triethylamine | ,258 | 6 | ,200^*^ | ,923 | 6 | ,526 |
| Buthylamine | ,172 | 6 | ,200^*^ | ,973 | 6 | ,913 |
| Benzaldehyde | ,266 | 6 | ,200^*^ | ,838 | 6 | ,126 |
| Beta citronellol | ,174 | 6 | ,200^*^ | ,943 | 6 | ,685 |
| Formic acid | ,281 | 6 | ,151 | ,890 | 6 | ,316 |
| Acetic acid (ac2) | ,193 | 6 | ,200^*^ | ,892 | 6 | ,328 |
| Propionic acid | ,210 | 6 | ,200^*^ | ,889 | 6 | ,315 |
| Butanoic acid | ,289 | 6 | ,128 | ,905 | 6 | ,407 |
| Hexanoic acid | ,225 | 6 | ,200^*^ | ,913 | 6 | ,458 |
| Octanoi acid | ,166 | 6 | ,200^*^ | ,968 | 6 | ,877 |
| Solvent - Water | ,269 | 6 | ,200^*^ | ,894 | 6 | ,340 |
| Solvent - Ethanol | ,195 | 6 | ,200^*^ | ,956 | 6 | ,791 |
| *. This is a lower bound of the true significance. | | | | | | |
| a. Lilliefors Significance Correction | | | | | | |

| **Tests of Normality -** *w;pUASDsuzIR75d^HEK^/pIR75d-Gal4;IR75^KO^* | | | | | | |
| --- | --- | --- | --- | --- | --- | --- |
|  | Kolmogorov-Smirnov^a^ | | | Shapiro-Wilk | | |
|  | Statistic | df | Sig. | Statistic | df | Sig. |
| Phenylacetaldehyde (ac4) | ,276 | 7 | ,115 | ,901 | 7 | ,339 |
| 3-octanol (ac3) | ,309 | 7 | ,042 | ,830 | 7 | ,080 |
| Pyridine (ac2) | ,166 | 7 | ,200^*^ | ,971 | 7 | ,905 |
| Putrescine (ac2) | ,234 | 7 | ,200^*^ | ,872 | 7 | ,193 |
| Pyrrolidine (ac1/ac2/ac4) | ,206 | 7 | ,200^*^ | ,902 | 7 | ,340 |
| Dimethylamine (ac1/ac4) | ,163 | 7 | ,200^*^ | ,903 | 7 | ,347 |
| Ammonium hydroxide (ac1/ac4) | ,180 | 7 | ,200^*^ | ,894 | 7 | ,294 |
| Phenylaceic acid (ac4) | ,208 | 7 | ,200^*^ | ,909 | 7 | ,387 |
| 2-phenyletylamine (ac1/ac4) | ,263 | 7 | ,156 | ,809 | 7 | ,051 |
| Ammonia (ac1/ac4) | ,215 | 7 | ,200^*^ | ,958 | 7 | ,799 |
| Cadaverine (ac2) | ,184 | 7 | ,200^*^ | ,946 | 7 | ,689 |
| Spermidine (ac2) | ,211 | 7 | ,200^*^ | ,895 | 7 | ,303 |
| Hexylamine | ,278 | 7 | ,108 | ,789 | 7 | ,032 |
| Triethylamine | ,247 | 7 | ,200^*^ | ,838 | 7 | ,096 |
| Buthylamine | ,244 | 7 | ,200^*^ | ,881 | 7 | ,231 |
| Benzaldehyde | ,266 | 7 | ,145 | ,869 | 7 | ,182 |
| Beta citronellol | ,316 | 7 | ,034 | ,820 | 7 | ,064 |
| Formic acid | ,235 | 7 | ,200^*^ | ,941 | 7 | ,649 |
| Acetic acid (ac2) | ,199 | 7 | ,200^*^ | ,924 | 7 | ,503 |
| Propionic acid | ,242 | 7 | ,200^*^ | ,941 | 7 | ,651 |
| Butanoic acid | ,282 | 7 | ,097 | ,847 | 7 | ,116 |
| Hexanoic acid | ,168 | 7 | ,200^*^ | ,984 | 7 | ,978 |
| Octanoi acid | ,174 | 7 | ,200^*^ | ,979 | 7 | ,953 |
| Solvent - Water | ,169 | 7 | ,200^*^ | ,930 | 7 | ,552 |
| Solvent - Ethanol | ,269 | 7 | ,134 | ,910 | 7 | ,392 |
| *. This is a lower bound of the true significance. | | | | | | |
| a. Lilliefors Significance Correction | | | | | | |

| **Tests of Normality -** *w;Bl/CyO;pIR84a-Gal4^KI^* | | | | | | |
| --- | --- | --- | --- | --- | --- | --- |
|  | Kolmogorov-Smirnov^a^ | | | Shapiro-Wilk | | |
|  | Statistic | df | Sig. | Statistic | df | Sig. |
| Phenylacetaldehyde (ac4) | ,157 | 7 | ,200^*^ | ,982 | 7 | ,970 |
| 3-octanol (ac3) | ,187 | 7 | ,200^*^ | ,948 | 7 | ,710 |
| Pyridine (ac2) | ,251 | 7 | ,200^*^ | ,890 | 7 | ,274 |
| Putrescine (ac2) | ,156 | 7 | ,200^*^ | ,939 | 7 | ,627 |
| Pyrrolidine (ac1/ac2/ac4) | ,210 | 7 | ,200^*^ | ,878 | 7 | ,218 |
| Dimethylamine (ac1/ac4) | ,168 | 7 | ,200^*^ | ,939 | 7 | ,633 |
| Ammonium hydroxide (ac1/ac4) | ,158 | 7 | ,200^*^ | ,967 | 7 | ,879 |
| Phenylaceic acid (ac4) | ,320 | 7 | ,029 | ,818 | 7 | ,061 |
| 2-phenyletylamine (ac1/ac4) | ,285 | 7 | ,089 | ,716 | 7 | ,006 |
| Ammonia (ac1/ac4) | ,244 | 7 | ,200^*^ | ,861 | 7 | ,154 |
| Cadaverine (ac2) | ,216 | 7 | ,200^*^ | ,892 | 7 | ,285 |
| Spermidine (ac2) | ,159 | 7 | ,200^*^ | ,953 | 7 | ,754 |
| Hexylamine | ,163 | 7 | ,200^*^ | ,957 | 7 | ,794 |
| Triethylamine | ,189 | 7 | ,200^*^ | ,956 | 7 | ,788 |
| Buthylamine | ,330 | 7 | ,021 | ,812 | 7 | ,054 |
| Benzaldehyde | ,153 | 7 | ,200^*^ | ,970 | 7 | ,897 |
| Beta citronellol | ,341 | 7 | ,013 | ,736 | 7 | ,009 |
| Formic acid | ,221 | 7 | ,200^*^ | ,878 | 7 | ,217 |
| Acetic acid (ac2) | ,265 | 7 | ,147 | ,899 | 7 | ,324 |
| Propionic acid | ,315 | 7 | ,034 | ,758 | 7 | ,015 |
| Butanoic acid | ,202 | 7 | ,200^*^ | ,903 | 7 | ,353 |
| Hexanoic acid | ,180 | 7 | ,200^*^ | ,932 | 7 | ,568 |
| Octanoi acid | ,208 | 7 | ,200^*^ | ,908 | 7 | ,379 |
| Solvent - Water | ,230 | 7 | ,200^*^ | ,918 | 7 | ,458 |
| Solvent - Ethanol | ,166 | 7 | ,200^*^ | ,933 | 7 | ,577 |
| *. This is a lower bound of the true significance. | | | | | | |
| a. Lilliefors Significance Correction | | | | | | |

| **Tests of Normality -** *w;pUASDsuzIR64a;pIR84a-Gal4^KI^* | | | | | | |
| --- | --- | --- | --- | --- | --- | --- |
|  | Kolmogorov-Smirnov^a^ | | | Shapiro-Wilk | | |
|  | Statistic | df | Sig. | Statistic | df | Sig. |
| Phenylacetaldehyde (ac4) | ,231 | 5 | ,200^*^ | ,909 | 5 | ,462 |
| 3-octanol (ac3) | ,304 | 5 | ,149 | ,817 | 5 | ,111 |
| Pyridine (ac2) | ,117 | 5 | ,200^*^ | ,995 | 5 | ,994 |
| Putrescine (ac2) | ,212 | 5 | ,200^*^ | ,906 | 5 | ,443 |
| Pyrrolidine (ac1/ac2/ac4) | ,206 | 5 | ,200^*^ | ,930 | 5 | ,595 |
| Dimethylamine (ac1/ac4) | ,219 | 5 | ,200^*^ | ,884 | 5 | ,326 |
| Ammonium hydroxide (ac1/ac4) | ,181 | 5 | ,200^*^ | ,966 | 5 | ,851 |
| Phenylaceic acid (ac4) | ,168 | 5 | ,200^*^ | ,978 | 5 | ,924 |
| 2-phenyletylamine (ac1/ac4) | ,298 | 5 | ,167 | ,848 | 5 | ,190 |
| Ammonia (ac1/ac4) | ,259 | 5 | ,200^*^ | ,857 | 5 | ,219 |
| Cadaverine (ac2) | ,229 | 5 | ,200^*^ | ,903 | 5 | ,429 |
| Spermidine (ac2) | ,201 | 5 | ,200^*^ | ,881 | 5 | ,314 |
| Hexylamine | ,294 | 5 | ,182 | ,860 | 5 | ,228 |
| Triethylamine | ,228 | 5 | ,200^*^ | ,875 | 5 | ,287 |
| Buthylamine | ,351 | 5 | ,044 | ,704 | 5 | ,010 |
| Benzaldehyde | ,231 | 5 | ,200^*^ | ,881 | 5 | ,314 |
| Beta citronellol | ,399 | 5 | ,009 | ,741 | 5 | ,025 |
| Formic acid | ,236 | 5 | ,200^*^ | ,876 | 5 | ,291 |
| Acetic acid (ac2) | ,266 | 5 | ,200^*^ | ,891 | 5 | ,364 |
| Propionic acid | ,196 | 5 | ,200^*^ | ,944 | 5 | ,697 |
| Butanoic acid | ,311 | 5 | ,128 | ,880 | 5 | ,309 |
| Hexanoic acid | ,208 | 5 | ,200^*^ | ,967 | 5 | ,859 |
| Octanoi acid | ,250 | 5 | ,200^*^ | ,872 | 5 | ,274 |
| Solvent - Water | ,265 | 5 | ,200^*^ | ,881 | 5 | ,314 |
| Solvent - Ethanol | ,286 | 5 | ,200^*^ | ,813 | 5 | ,103 |
| *. This is a lower bound of the true significance. | | | | | | |
| a. Lilliefors Significance Correction | | | | | | |

| **Tests of Normality -** *w;pUASCpomIR64a∆;pIR84a-Gal4^KI^* | | | | | | |
| --- | --- | --- | --- | --- | --- | --- |
|  | Kolmogorov-Smirnov^a^ | | | Shapiro-Wilk | | |
|  | Statistic | df | Sig. | Statistic | df | Sig. |
| Phenylacetaldehyde (ac4) | ,266 | 6 | ,200^*^ | ,886 | 6 | ,300 |
| 3-octanol (ac3) | ,172 | 6 | ,200^*^ | ,976 | 6 | ,930 |
| Pyridine (ac2) | ,256 | 6 | ,200^*^ | ,923 | 6 | ,531 |
| Putrescine (ac2) | ,265 | 6 | ,200^*^ | ,910 | 6 | ,435 |
| Pyrrolidine (ac1/ac2/ac4) | ,242 | 6 | ,200^*^ | ,940 | 6 | ,661 |
| Dimethylamine (ac1/ac4) | ,316 | 6 | ,062 | ,833 | 6 | ,114 |
| Ammonium hydroxide (ac1/ac4) | ,238 | 6 | ,200^*^ | ,893 | 6 | ,332 |
| Phenylaceic acid (ac4) | ,274 | 6 | ,177 | ,890 | 6 | ,320 |
| 2-phenyletylamine (ac1/ac4) | ,181 | 6 | ,200^*^ | ,922 | 6 | ,520 |
| Ammonia (ac1/ac4) | ,236 | 6 | ,200^*^ | ,900 | 6 | ,374 |
| Cadaverine (ac2) | ,193 | 6 | ,200^*^ | ,938 | 6 | ,642 |
| Spermidine (ac2) | ,195 | 6 | ,200^*^ | ,910 | 6 | ,439 |
| Hexylamine | ,221 | 6 | ,200^*^ | ,958 | 6 | ,804 |
| Triethylamine | ,178 | 6 | ,200^*^ | ,936 | 6 | ,629 |
| Buthylamine | ,292 | 6 | ,121 | ,872 | 6 | ,236 |
| Benzaldehyde | ,135 | 6 | ,200^*^ | ,995 | 6 | ,998 |
| Beta citronellol | ,245 | 6 | ,200^*^ | ,795 | 6 | ,053 |
| Formic acid | ,196 | 6 | ,200^*^ | ,879 | 6 | ,263 |
| Acetic acid (ac2) | ,184 | 6 | ,200^*^ | ,950 | 6 | ,740 |
| Propionic acid | ,267 | 6 | ,200^*^ | ,878 | 6 | ,261 |
| Butanoic acid | ,235 | 6 | ,200^*^ | ,908 | 6 | ,420 |
| Hexanoic acid | ,280 | 6 | ,154 | ,922 | 6 | ,519 |
| Octanoi acid | ,281 | 6 | ,152 | ,869 | 6 | ,223 |
| Solvent - Water | ,277 | 6 | ,165 | ,887 | 6 | ,305 |
| Solvent - Ethanol | ,297 | 6 | ,108 | ,864 | 6 | ,203 |
| *. This is a lower bound of the true significance. | | | | | | |
| a. Lilliefors Significance Correction | | | | | | |

DsuzIR64a neuronal counting

| **Tests of Normality** | | | | | | |
| --- | --- | --- | --- | --- | --- | --- |
|  | Kolmogorov-Smirnov^a^ | | | Shapiro-Wilk | | |
|  | Statistic | df | Sig. | Statistic | df | Sig. |
| Male | ,249 | 7 | ,200^*^ | ,893 | 7 | ,292 |
| Female | ,150 | 7 | ,200^*^ | ,960 | 7 | ,815 |
| *. This is a lower bound of the true significance. | | | | | | |
| a. Lilliefors Significance Correction | | | | | | |

| **Independent Samples Test** | | | | | |
| --- | --- | --- | --- | --- | --- |
|  | | Levene's Test for Equality of Variances | | t-test for Equality of Means | |
|  |  | F | Sig. | t | df |
|  |  |  |  |  |  |
| Neurons | Equal variances assumed | 4,404 | ,049 | 1,252 | 20 |
|  | Equal variances not assumed |  |  | 1,018 | 7,776 |

| **Independent Samples Test** | | | | |
| --- | --- | --- | --- | --- |
|  | | t-test for Equality of Means | | |
|  |  | Significance | | Mean Difference |
|  |  | One-Sided p | Two-Sided p |  |
| Neurons | Equal variances assumed | ,113 | ,225 | 4,21905 |
|  | Equal variances not assumed | ,170 | ,339 | 4,21905 |

| **Independent Samples Test** | | | | |
| --- | --- | --- | --- | --- |
|  | | t-test for Equality of Means | | |
|  |  | Std. Error Difference | 95% Confidence Interval of the Difference | |
|  |  |  | Lower | Upper |
| VAR00002 | Equal variances assumed | 3,37046 | -2,81160 | 11,24970 |
|  | Equal variances not assumed | 4,14618 | -5,39012 | 13,82822 |
