# Supplementary material for: Alternative strategies based on transgenic Drosophila melanogaster for the functional characterization of insect Ionotropic Receptors
Source: Biol Res. 2025 Jun 9;58:36. doi: 10.1186/s40659-025-00619-0 (PMC12147327; doi:10.1186/s40659-025-00619-0)
Supplement: Supplementary file 10 — Supplementary file 4. Figure S1. Schematic representation of crossings to generate transgenic lines for SSR. Note: for the various transgenic lines, the transgene is indicated in bold [file 40659_2025_619_MOESM10_ESM.pdf]

CpomIR41a lines

w ; pIR76a-Gal4 ; $\frac{TM2}{TM6B}$ X *w ;* $\frac{Bl}{CyO}$ *; pUAS-**CpomIR41a1*** → *w ; pIR76a-Gal4 ; pUAS-**CpomIR41a1***

DsuzIR75d^{HEK} lines

w ; pIR75d-Gal4 ; $\frac{TM2}{TM6B}$ X *w ;* $\frac{Bl}{CyO}$ *;* $IR75d^{KO}$ → *w ; pIR75d-Gal4 ;* $IR75d^{KO}$

X → *w ;* $\frac{pIR75d\text{-}Gal4}{pUAS\text{-}\mathbf{DsuzIR75d}^{HEK}}$ *;* $IR75d^{KO}$

*w ; pUAS-**DsuzIR75d***^{HEK} *;* $\frac{TM2}{TM6B}$ X *w ;* $\frac{Bl}{CyO}$ *;* $IR75d^{KO}$ → *w ; pUAS-**DsuzIR75d***^{HEK} *;* $IR75d^{KO}$

Dsuz/CpomIR64a lines

*w ; pUAS-**(Dsuz/Cpom)IR64a** ;* $\frac{TM2}{TM6B}$ X *w ;* $\frac{Bl}{CyO}$ *;* $pIR84aGal4^{KI}$ → *w ; pUAS-**(Dsuz/Cpom)IR64a** ;* $pIR84aGal4^{KI}$
