# Supplementary material for: Alternative strategies based on transgenic Drosophila melanogaster for the functional characterization of insect Ionotropic Receptors
Source: Biol Res. 2025 Jun 9;58:36. doi: 10.1186/s40659-025-00619-0 (PMC12147327; doi:10.1186/s40659-025-00619-0)

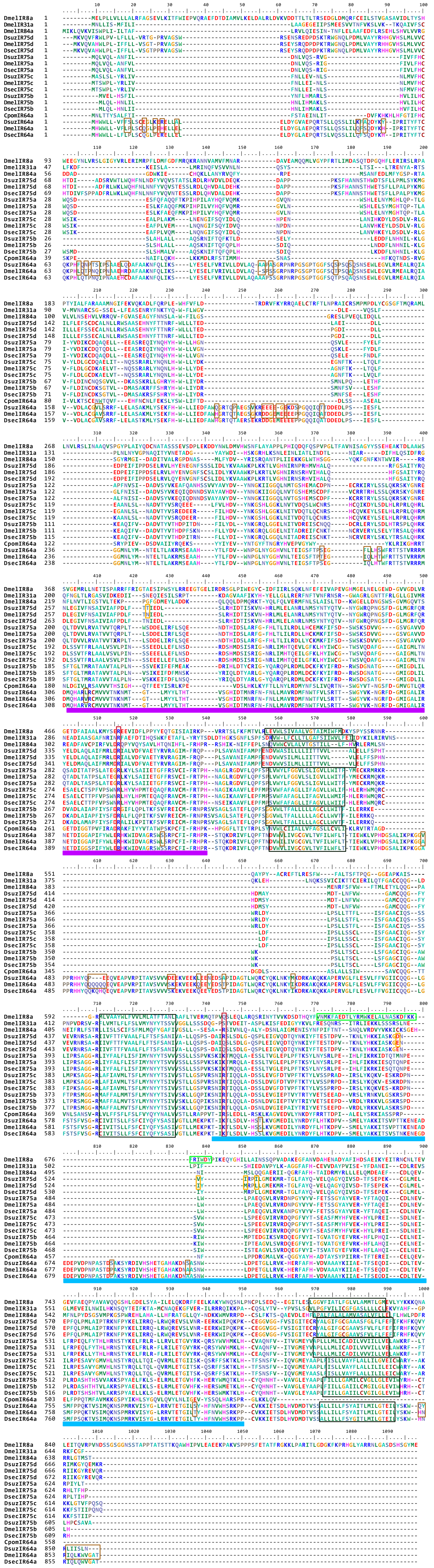

Supplement: Supplementary file 1 — Supplementary file 7. Figure S4. Supplementary polypeptide sequence alignment of IR75d orthologues together with acid sensing subunits. In the dataset we included the following: DsuzIR75a; DsuzIR75b [33]; DsuzIR75c; DsuzIR75d and DsuzIR64a; DmelIR75b; DmelIR75c; DmelIR75d; DmelIR64a; DmelIR84a; DmelIR31a Isoform C; DmelIR8a; DsecIR75a; DsecIR75b; DsecIR75c; DsecIR75d; DsecIR64a; CpomIR64a. Sequences were aligned using Muscle software. Alignment accuracy was checked manually and refined using BioEdit v7.2.5 [44]. Lilac filled square: S1 domain; light-blue filled square: S2 domains, according to Prieto-Godino et al. [21]. Green square: S2-CREL according with Abuin et al. [9]. Note: Prieto-Godino et al. [21] used a variant of DmelIR75b, which is 4 amino acids longer and no PDB-files are available for this variant. Black squares: transmembrane domains based on TopCons. Orange squares: amino acid substitutions identified within the LBD of DmelIR75d, when compared with the D. suzukii orthologue. Brown squares: amino acid substitutions, insertions and mutations between the IR64a orthologues of D. melanogaster and D. suzukii. Red square: arginine proximal to the end of the LBD-S1 domain, which is conserved in iGluRs and acid-sensing IRs, assigned for carboxyl group binding [12, 42]. With the red square we have also highlighted an additional positively-charged residue we identified at the beginning of the LBD-S2 domain, which is conserved in all acid sensing IRs, except for IR31a. [file 40659_2025_619_MOESM1_ESM.pdf]
